# Tissue metabolites in diffuse glioma and their modulations by *IDH1* mutation, histology, and treatment

**DOI:** 10.1172/jci.insight.153526

**Published:** 2022-02-08

**Authors:** Christoph Trautwein, Laimdota Zizmare, Irina Mäurer, Benjamin Bender, Björn Bayer, Ulrike Ernemann, Marcos Tatagiba, Stefan J. Grau, Bernd J. Pichler, Marco Skardelly, Ghazaleh Tabatabai

**Affiliations:** 1 Department of Preclinical Imaging and Radiopharmacy, Werner Siemens Imaging Center,; 2 Department of Neurology & Interdisciplinary Neuro-Oncology,; 3 Center for Neuro-Oncology, Comprehensive Cancer Center Tübingen,; 4 Department of Neuroradiology, and; 5 Department of Neurosurgery, University Hospital Tübingen, Eberhard Karls University Tübingen, Tübingen, Germany.; 6 Center for Neurosurgery, Department of Neurosurgery, University Hospital Cologne, Cologne, Germany.; 7 Cluster of Excellence, EXC 2180, Image Guided and Functionally Instructed Tumor Therapies, University Hospital Tübingen, Eberhard Karls University of Tübingen, Tübingen, Germany.; 8 German Cancer Consortium (DKTK), Deutsches Krebs Forschungszentrum partner site Tübingen, Tübingen, Germany.

**Keywords:** Oncology, Brain cancer

## Abstract

The discovery of the oncometabolite 2-hydroxyglutarate in isocitrate dehydrogenase 1–mutated (*IDH1*-mutated) tumor entities affirmed the role of metabolism in cancer. However, large databases with tissue metabolites that are modulated by *IDH1* mutation remain an area of development. Here, we present an unprecedented and valuable resource for tissue metabolites in diffuse glioma and their modulations by *IDH1* mutation, histology, and tumor treatments in 101 tissue samples from 73 diffuse glioma patients (24 astrocytoma, 17 oligodendroglioma, 32 glioblastoma), investigated by NMR-based metabolomics and supported by RNA-Seq. We discovered comparison-specific metabolites and pathways modulated by *IDH1* (*IDH1* mutation status cohort) and tumor entity. The Longitudinal investigation cohort provides metabolic profiles of untreated and corresponding treated glioma samples at first progression. Most interestingly, univariate and multivariate cox regressions and Kaplan-Meier analyses revealed that tissue metabolites correlate with progression-free and overall survival. Thus, this study introduces potentially novel candidate prognostic and surrogate metabolite biomarkers for future prospective clinical studies, aiming at further refining patient stratification in diffuse glioma. Furthermore, our data will facilitate the generation of so-far–unanticipated hypotheses for experimental studies to advance our molecular understanding of glioma biology.

## Introduction

Mutations in isocitrate dehydrogenase 1 (*IDH1*) or *IDH2* are frequent in several cancer entities, including diffuse glioma (1–3). They lead to mutated enzymes with neomorphic activity, producing the oncometabolite 2-hydroxyglutarate (2-HG), which modulates cellular epigenetic programs, differentiation patterns, and metabolic profiles (4–6). Recent clinical trials in diffuse glioma have used the *IDH1* mutation (*IDH1*-mut) as a therapeutic target (7, 8). However, the therapeutic efficacy of these therapeutic approaches might critically depend on understanding metabolic alterations that occur in the context of *IDH1* mutations. For example, 2-HG shapes the tumor immune microenvironment by suppressing T cell activity in glioma (9). Furthermore, the nucleotide synthesis utilization and DNA repair capacity is different in *IDH1*-mut and *IDH1*-WT glioma (10). These observations indicate that *IDH1*-mut glioma exhibit distinct therapeutic challenges and mechanisms for resistance to therapy.

Altered metabolism is a major hallmark of cancer, with the potential to promote tumor growth and acquired resistance to therapy (11). Glioma cells display abnormal energy metabolism, including alterations of glucose, amino acid, and fatty acid metabolism (12). Altered metabolic pathways including choline, taurine, hypotaurine, or glutamate/glutamine correlate with different WHO grades in diffuse glioma (13). Noninvasive detection of glutamate by magnetic resonance spectroscopy can serve as a metabolic imaging biomarker of response to temozolomide treatment in *IDH1*-mut glioma (14).

## Results

### Study cohorts and comparisons based on clinical characteristics.

**Figure 1:**
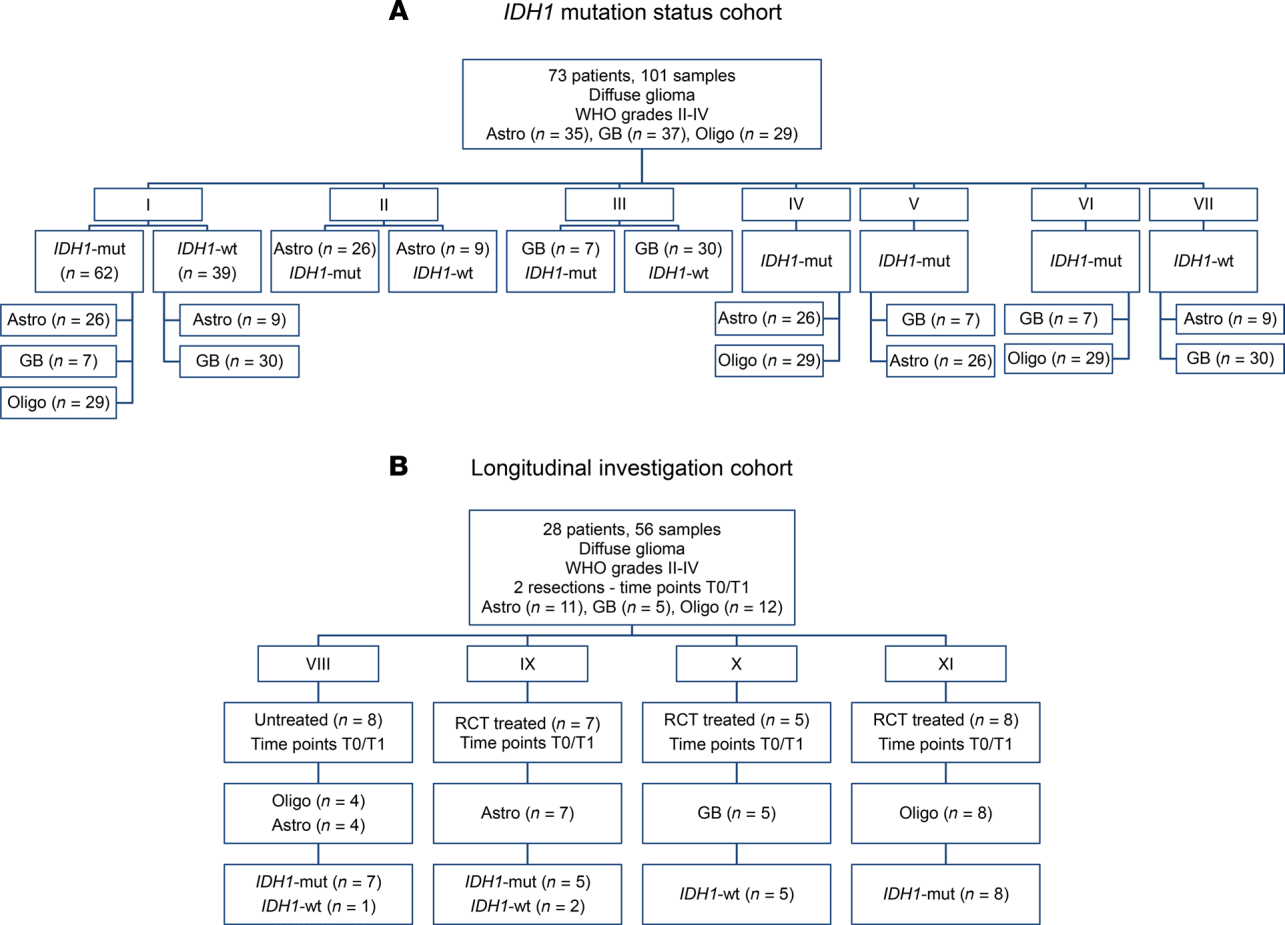
Overview of study cohort and comparisons. We created 2 cohorts with distinct comparisons in each cohort. (**A**) *IDH1* mutation status cohort (with comparisons I, II, III, IV, V, VI, VII). (**B**) The Longitudinal investigation cohort (with comparisons VIII, IX, X, XI). The number of patients and samples in each comparison are indicated. Astro, astrocytoma; GB, glioblastoma; *IDH1-*mut*,* *IDH1* mutation; Oligo, oligodendroglioma; RCT, radio- and/or chemotherapy.

We investigated distinct comparisons of tissue metabolites derived from NMR analyses, complemented with gene expression data from the RNA-Seq in selected comparisons, to investigate modulations by the *IDH1* mutation and histology in the *IDH1* mutation status cohort (Figure 1A), and we examined tumor treatments in tissue pairs from newly diagnosed and corresponding progressive glioma with or without treatment between resections in the Longitudinal investigation cohort (Figure 1B).

In the *IDH1* mutation status cohort (Figure 1A), we compared *n* = 62 *IDH1-*mut with *n* = 39 *IDH1-*WT samples (Figure 1A, comparison I), *n* = 26 *IDH1*-mut astrocytoma with *n* = 9 *IDH1-*WT astrocytoma (Figure 1A, comparison II), and *n* = 7 *IDH1-*mut glioblastoma with *n* = 30 *IDH1-*WT glioblastoma (Figure 1A, comparison III) upon differences in metabolites, pathways, and gene expression profiles. Additionally, we included further comparisons in the *IDH1* mutation status cohort investigating metabolite and pathway alterations in *n* = 26 *IDH1*-mut astrocytoma versus *n* = 29 oligodendroglioma (Figure 1A, comparison IV), *n* = 7 *IDH1*-mut glioblastoma versus *n* = 26 *IDH1*-mut astrocytoma (Figure 1A, comparison V), *n* = 7 *IDH1*-mut glioblastoma versus *n* = 29 oligodendroglioma (Figure 1A, comparison VI), and *n* = 9 *IDH1*-WT astrocytoma versus *n* = 30 *IDH1*-WT glioblastoma (Figure 1A, comparison VII).

Finally, in the Longitudinal investigation cohort (Figure 1B), we compared tissues from newly diagnosed (time point 0 [T0]) and corresponding progressive glioma (T1) either without (untreated) or with treatment (treated) between both resections. We pooled all tumor therapies in the category “treated.” The comparisons in this cohort included: *n* = 4 astrocytoma (*n* = 3 *IDH1*-mut, *n* = 1 *IDH1*-WT) and *n* = 4 oligodendroglioma with confirmed *IDH1* mutation and 1p/19q codeletion (*IDH1*-mut) without any treatment between resections (Figure 1B, comparison VIII); *n* = 7 astrocytoma (5 *IDH1*-mut, 2 *IDH1*-WT) patients who received treatment between resections (Figure 1B, comparison IX); *n* = 5 *IDH1*-WT glioblastoma patients (Figure 1B, comparison X) who were treated between resections; and *n* = 8 oligodendroglioma with confirmed *IDH1* mutation and 1p/19q codeletion upon treatment between both resections (Figure 1B, comparison XI).

### IDH1 mutation leads to a distinct metabolite profile, pathway alterations, and gene expression patterns in astrocytoma, oligodendroglioma, and glioblastoma.

**Figure 2:**
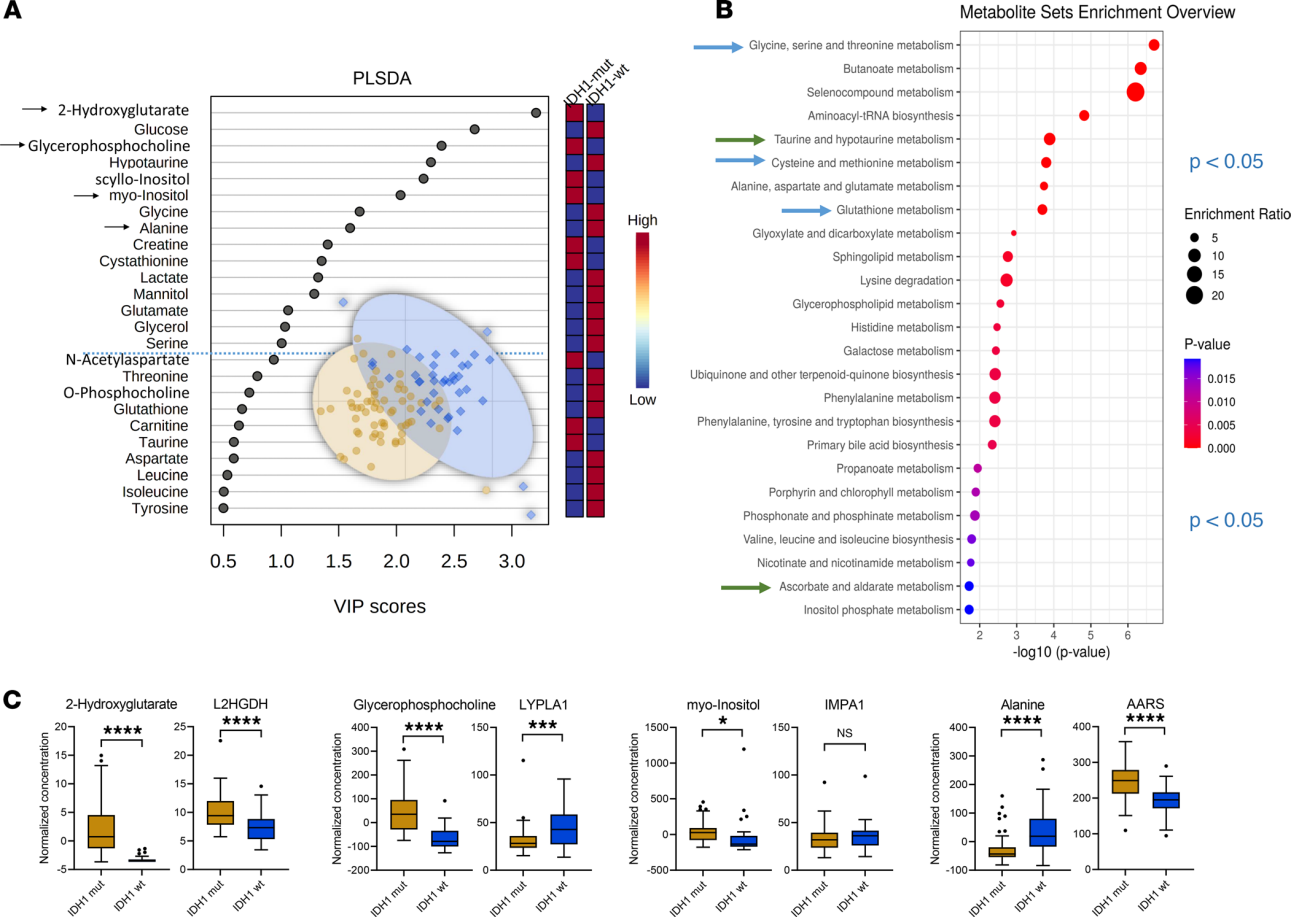
*IDH1* mutation status cohort comparison I. (**A**–**C**) Multivariate partial least squares–discriminant analysis (PLS-DA) identifies 15 metabolites with variable importance in projection (VIP) scores > 1 (**A**) and more than 25 altered pathways as obtained by quantitative enrichment analysis (**B**) in the *IDH1-*mutation (*IDH1-*mut, *n* = 62) versus WT (*IDH1-*WT, *n* = 39), selection of significant metabolites (as indicated by arrow in the VIP scores plot), and their corresponding gene expression changes (**C**) in the *IDH1* mutation status cohort comparison (I) illustrated as box-and-whisker plot with Tukey’s range test. ****P < 0.0001, ***P < 0.001, *P < 0.05, determined by unpaired parametric 2-tailed *t* test with Welch’s correction. Illustrated as box plots with whiskers based on Tukey range test, showing outliers. Arrows in VIP scores plots indicate metabolites that were selected and correlated with the RNA-Seq data. Blue dotted line indicates the PLS-DA VIP score 1.0 threshold cut-off. Arrows in metabolite set enrichment overview represent most interesting and relevant metabolic pathway changes. L2HGDH, L-2-hydroxyglutarate dehydrogenase; AARS, alanyl-tRNA synthetase; LYPLA1, lysophospholipase 1; IMPA1, inositol monophosphatase 1.

By comparison I (*IDH1*-mut [*n* = 62] versus *IDH1*-WT [*n* = 39]) in the *IDH1* mutation status cohort (Figure 1A), we detected 26 altered metabolites by univariate statistical testing with P < 0.05 and fold change > 1.2 including 2-HG, sn-glycero-3-phosphocholine, scyllo-inositol, alanine, myo-inositol, hypotaurine, sarcosine, cystathionine, glutathione, and mannitol (Table 1 and Table 2) and 15 partial least squares–discriminant analysis (PLS-DA) variables with a variable importance in projection (VIP) score greater than 1 (Figure 2A). When applying quantitative metabolite enrichment analysis for altered pathways within our *IDH1* mutation status cohort, we identified more than 25 significant (*P* < 0.05) pathways in comparison I (Figure 2B). We complemented the following top-scored metabolites from the receiver operating characteristics (ROC) analysis by correlating them with gene expression levels from the RNA-Seq data (Supplemental Figure 1 and Supplemental Table 1; supplemental material available online with this article; https://doi.org/10.1172/jci.insight.153526DS1): 2-HG with L-2-HG dehydrogenase (L2HGDH), alanine with alanyl-tRNA synthetase (AARS), sn-glycero-3-phosphocholine with lysophospholipase 1 (LYPLA1), and myo-inositol with inositol monophosphatase 1 (IMPA1) (Figure 2C).

**Figure 3:**
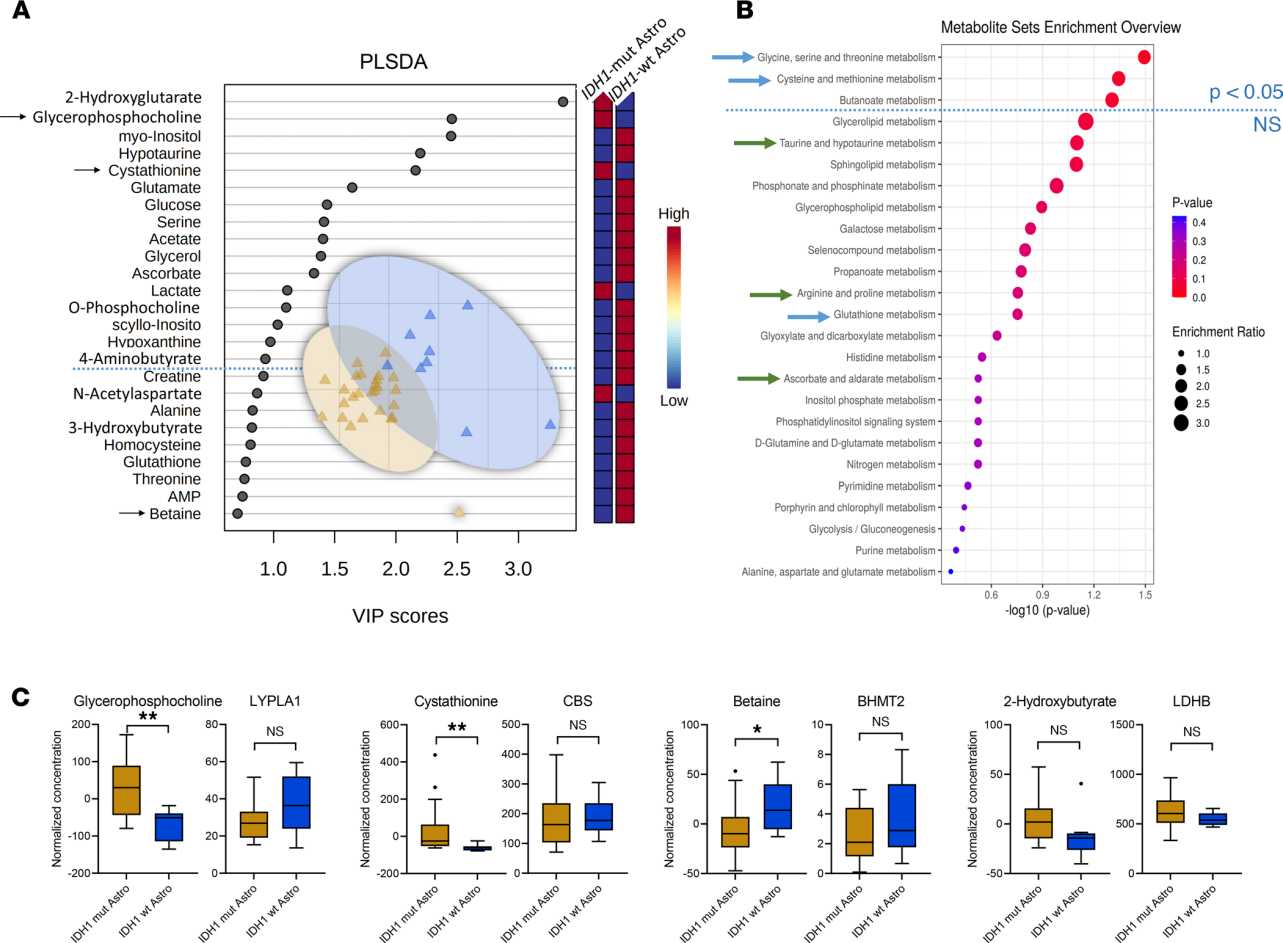
*IDH1* mutation status cohort comparisons II. (**A**–**C**) Multivariate partial least squares–discriminant analysis (PLS-DA) identifies 16 metabolites with variable importance in projection (VIP) scores > 1 (**A**) and only 3 altered pathways by quantitative enrichment analysis (**B**) for comparing *IDH1*-mut (*n* = 26) versus WT (*n* = 9) of astrocytoma (Astro) subgroup (**A** and **B**, comparison II), selection of significant metabolites (as indicated by arrow in the VIP scores plot), and their corresponding gene expression changes (**C**) in the *IDH1* mutation status cohort comparison (II) illustrated as box-and-whisker plot with Tukey’s range test. **P < 0.01, *P < 0.05, determined by unpaired parametric 2-tailed *t* test with Welch’s correction. LYPLA1, lysophospholipase 1; *CBS*, cystathionine β-synthase; *BHMT2*, betaine-homocysteine S-methyltransferase 2; *LDHB*, lactate dehydrogenase B.

Comparison II (*IDH1*-mut astrocytoma [*n* = 26] versus *IDH1*-WT astrocytoma [*n* = 9]) (Figure 1A) identified sn-glycero-3-phosphocholine, hypoxanthine, betaine, and 2-HG as differentiating metabolites with *P* < 0.05 and fold change > 1.2 (Tables 1 and 2) and 16 PLS-DA variables (2-HG, cystathionine, sn-glycero-3-phosphocholine, betaine, histamine, 2-hydroxybutyrate, valine, hypoxanthine, ADP, and o-phosphocholine) with a VIP score > 1 (Figure 3A). In *IDH1*-mut versus *IDH1*-WT astrocytoma WHO grades II/III (comparison II), only 3 pathways were significantly (*P* < 0.05) altered (Figure 3B). We observed the following correlations of top-scored metabolite biomarkers from ROC analysis with changes in gene expression levels (Supplemental Figure 1B and Supplemental Table 1): cystathionine with cystathionine β-synthase (*CBS*), betaine with betaine-homocysteine S-methyltransferase 2 (*BHMT2*), 2-hydroxybutyrate with lactate dehydrogenase B (*LDHB*), and sn-glycero-3-phosphocholine with lysophospholipase 1 (*LYPLA1*) (Figure 3C).

**Figure 4:**
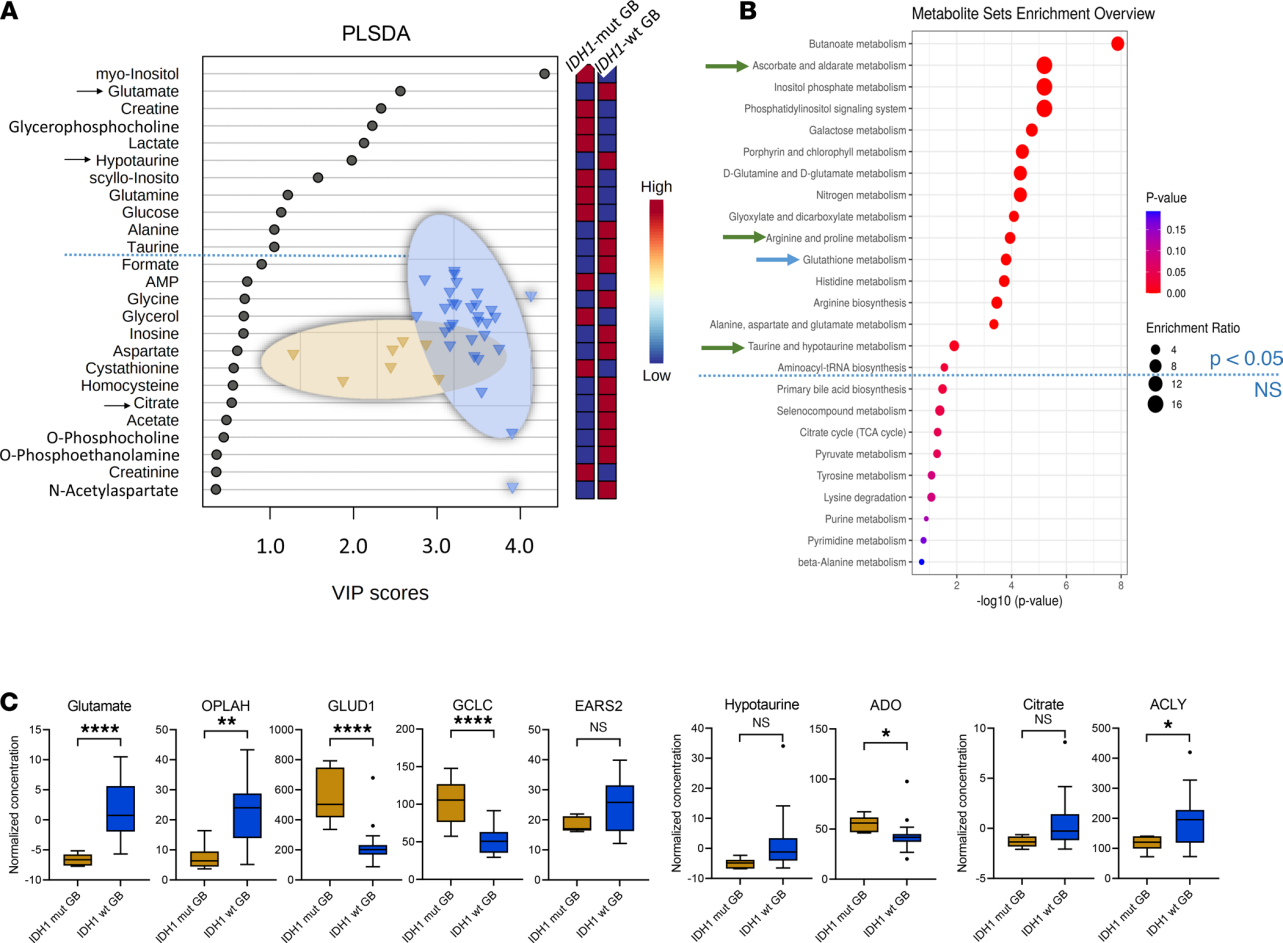
*IDH1* mutation status cohort comparison III. (**A**–**C**) PLS-DA identifies 11 metabolites with VIP scores > 1 (**A**) and 16 altered pathways (**B**) for comparing *IDH1*-mut (*n* = 7) versus WT (*n* = 30) glioblastoma (GB) subgroup (comparison III), selection of significant metabolites (as indicated by arrow in the VIP scores plot), and their corresponding gene expression changes (**C**) in the *IDH1* mutation status cohort comparison (III) illustrated as box-and-whisker plot with Tukey’s range test. ****P < 0.0001, **P < 0.01, *P < 0.05, determined by unpaired parametric 2-tailed *t* test with Welch’s correction. Arrows in VIP scores plots indicate metabolites that were selected and correlated with the RNA-Seq data. Blue dotted line indicates the PLS-DA VIP score 1.0 threshold cut-off. Arrows in metabolite set enrichment overview represent most interesting and relevant metabolic pathway changes. *OPLAH*, 5-oxoprolinase ATP-hydrolyzing; *GLUD1*, glutamate dehydrogenase 1; *GCLC*, glutamate-cysteine ligase catalytic subunit; *EARS2*, mitochondrial glutamyl-TRNA synthetase 2; *GOT1*, glutamic-oxaloacetic transaminase 1; *ACLY*, ATP citrate lyase; *ADO*, 2-aminoethanethiol dioxygenase.

By comparison III (*IDH1*-mut versus *IDH1*-WT glioblastoma) (Figure 1A, comparison III), we discovered 9 metabolites (2-HG, AMP, ATP, creatine, glutamate, taurine, myo-inositol, scyllo-inositol, and sn-glycero-3-phosphocholine) (*P* < 0.05 and fold change > 1.2) (Tables 1 and 2) and 11 PLS-DA variables with a VIP score > 1 (Figure 4A). When comparing *IDH1*-mut versus *IDH1*-WT glioblastoma (comparison III), we identified 16 pathway changes (Figure 4B). We compared our top-scored ROC biomarkers with alterations of the following gene expression levels (Supplemental Figure 1C and Supplemental Table 1): glutamate with 5-oxoprolinase ATP-hydrolysing (*OPLAH*), glutamate dehydrogenase 1 (*GLUD1*), glutamate-cysteine ligase catalytic subunit (*GCLC*), mitochondrial glutamyl-TRNA synthetase 2 (*EARS2*), and glutamic-oxaloacetic transaminase 1 (*GOT1*) and citrate with ATP citrate lyase (*ACYL*) and hypotaurine with 2-aminoethanethiol dioxygenase (*ADO*) (Figure 4C).

### Histology-dependent alterations of metabolites and pathways in diffuse glioma.

**Figure 5:**
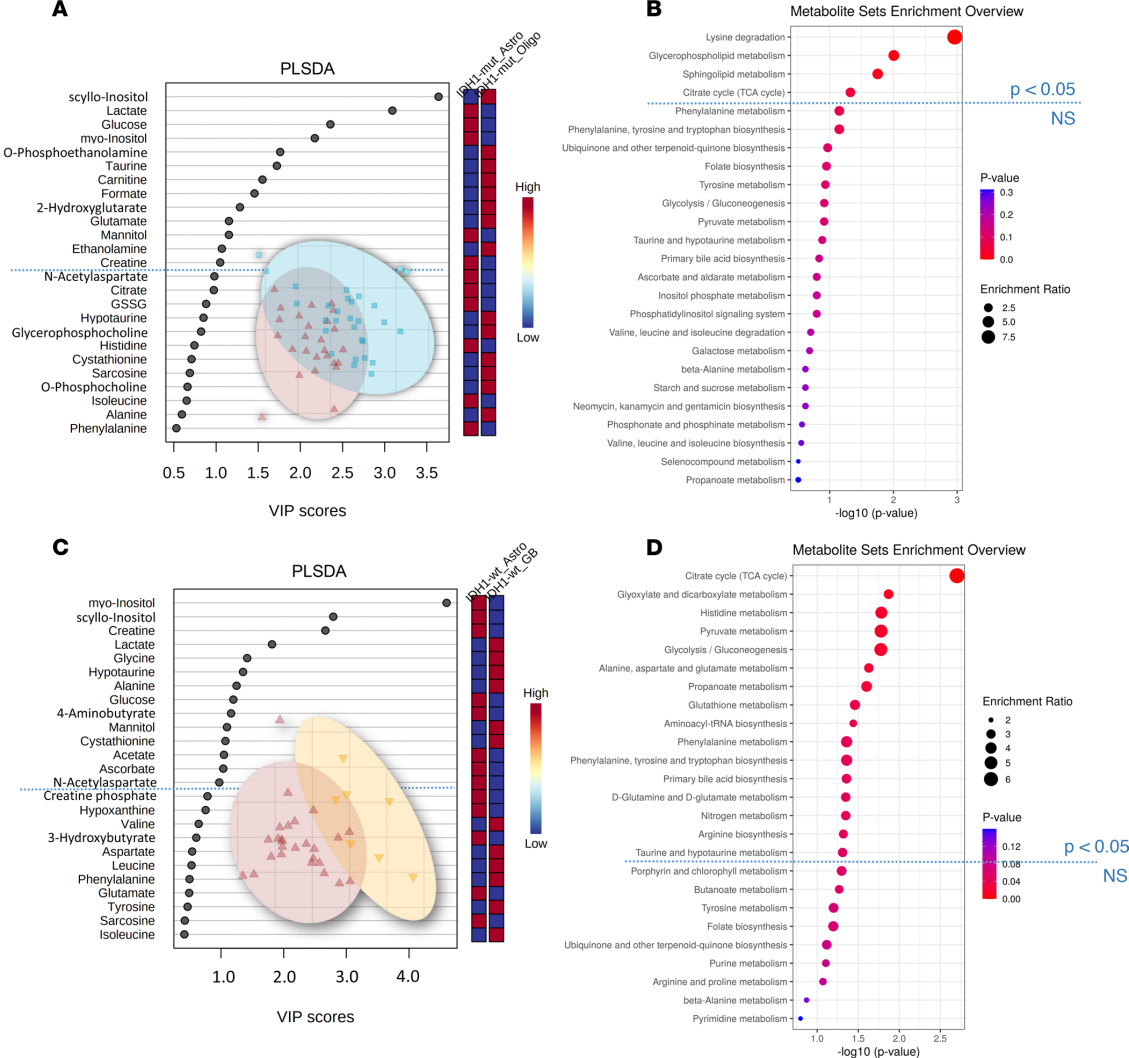
Histology-dependent cohort comparisons IV and V. (**A** and **B**) Partial least squares–discriminant analysis (PLS-DA) identifies 13 metabolites with variable importance in projection (VIP) scores > 1 (**A**) and 4 altered pathways by quantitative enrichment analysis (**B**) in the Astro *IDH1*-mut (*n* = 26) versus Oligo *IDH1*-mut (*n* = 29) comparison (IV). (**C** and **D**) Furthermore, PLS-DA identifies 14 metabolites with VIP scores > 1 (**C**) and 16 altered pathways (**D**) for comparing Astro *IDH1*-mut (*n* = 26) versus GB *IDH1*-mut (*n* = 7) (**C** and **D**, comparison V). Blue dotted line indicates the PLS-DA VIP score 1.0 threshold cut-off and significant metabolic pathway threshold (*P* < 0.05). Astro, astrocytoma; GB, glioblastoma; Oligo, oligodendroglioma.

Next, we performed comparisons between different histological subtypes with identical *IDH1* status. The comparison IV (Figure 1A) of *n* = 26 *IDH1*-mut astrocytoma versus *n* = 29 *IDH1*-mut oligodendroglioma identified 12 significant metabolite changes in the 2-tailed *t* test (Tables 1 and 2) (carnitine, citrate, ethanolamine, glutathione disulfide, histidine, mannitol, nicotinurate, o-acetylcholine, o-phosphoethanolamine, sarcosine, taurine, and scyllo-inositol). Furthermore, 13 PLS-DA metabolites with VIP scores > 1 (Figure 5A) and 4 statistically significant pathways (lysine degradation, glycerophospholipid metabolism, sphingolipid metabolism, and TCA cycle) were found (Figure 5B).

The comparison V (Figure 1A) of *n* = 26 *IDH1*-mut astrocytoma WHO grades II/III with *n* = 7 *IDH1*-mut glioblastoma (WHO grade IV) uncovered 15 significant metabolites in the 2-tailed *t* test (Tables 1 and 2), including ATP, butyrate, citrate, formate, fumarate, glutamate, glutathione, histamine, histidine, lactate, o-acetylcholine, phenylalanine, succinate, taurine, and uridine. Furthermore, 14 PLS-DA metabolites with VIP scores > 1 (Figure 5C) and 16 significant pathways (Figure 5D) were found (ascorbate and aldarate metabolism, inositol phosphate metabolism, phosphatidylinositol signaling system, arginine and proline metabolism, glycine, serine, and threonine metabolism).

**Figure 6:**
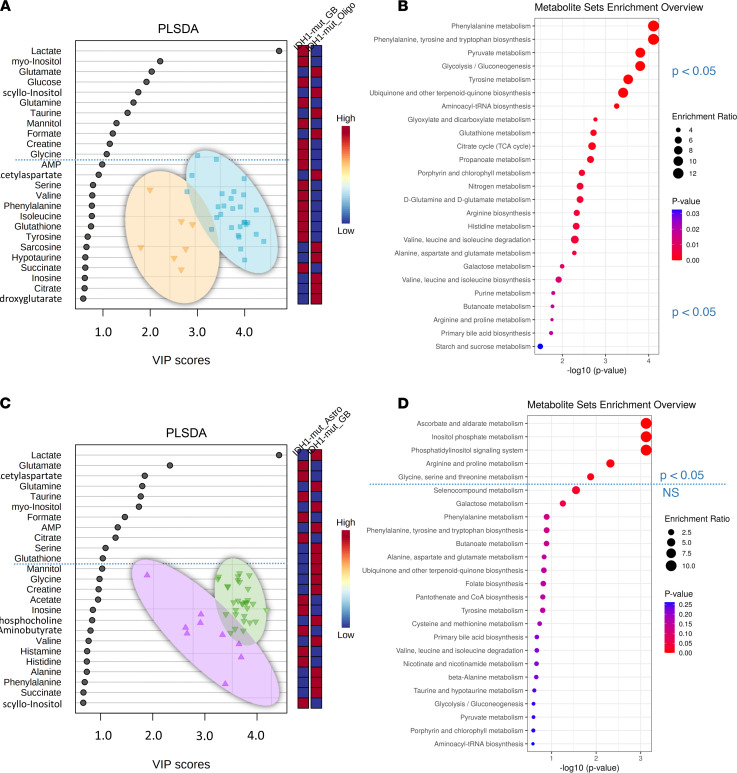
Histology-dependent cohort comparisons VI and VII. (**A**–**D**) Partial least squares–discriminant analysis (PLS-DA) with variable importance in projection (VIP) scores diagrams (**A** and **C**) and metabolite pathway enrichment overview (**B** and **D**) of GB *IDH1*-mut (*n* = 7) versus Oligo *IDH1*-mut (*n* = 29) (**A** and **B**, comparison VI) and Astro *IDH1*-WT (*n* = 9) versus GB *IDH1*-WT (*n* = 30) (**C** and **D**, comparison VII). Blue dotted line indicates the PLS-DA VIP score 1.0 threshold cut-off and significant metabolic pathway threshold (*P* < 0.05). Astro, astrocytoma; GB, glioblastoma; Oligo, oligodendroglioma.

In comparison VI (Figure 1A) (*n* = 7 *IDH1*-mut glioblastoma versus *n* = 29 *IDH1*-mut oligodendroglioma), we found 19 significant metabolites in the 2-tailed *t* test (Tables 1 and 2), including sarcosine, glutamate, taurine, AMP, uridine, phenylalanine, lactate, glutathione, formate, and citrate. Eleven metabolites with VIP scores > 1 were identified in the PLS-DA (Figure 6A), and more than 25 pathways, including phenylalanine metabolism, phenylalanine/tyrosine and tryptophan biosynthesis, pyruvate metabolism, glycolysis/gluconeogenesis, and tyrosine metabolism (Figure 6B), were found.

By comparison VII (Figure 1A) (*n* = 9 *IDH1*-WT astrocytoma WHO grades II/III versus *n* = 30 *IDH1*-WT glioblastoma), we identified 9 significant metabolites in the 2-tailed *t* test (4-aminobutyrate, alanine, ascorbate, butyrate, creatine, creatine phosphate, hypoxanthine, myo-inositol, and scyllo-inositol) (Tables 1 and 2) and 11 PLS-DA variables with VIP scores > 1 (Figure 6C). Quantitative enrichment analysis detected 5 significant pathways, including top 5 TCA cycle, glyoxylate and dicarboxylate metabolism, histidine metabolism, pyruvate metabolism, and glycolysis/gluconeogenesis (Figure 6D).

Furthermore, we performed ROC analysis of the top 4 significant metabolites in the histology-dependent comparisons (Supplemental Figures 2 and 3) and detected carnitine, o-phosphoethanolamine, ethanolamine, and histidine for comparison IV (Supplemental Figure 2A); citrate, glutathione, histamine, and formate for comparison V (Supplemental Figure 2B); sarcosine, glutamate, taurine, and AMP for comparison VI (Supplemental Figure 3A); and scyllo-inositol, 4-aminobutyrate, alanine, and betaine for comparison VI (Supplemental Figure 3B). The PLS-DA score plots of all comparisons I–VII are illustrated in Supplemental Figure 5.

### Longitudinal metabolic changes in glioma.

**Figure 7:**
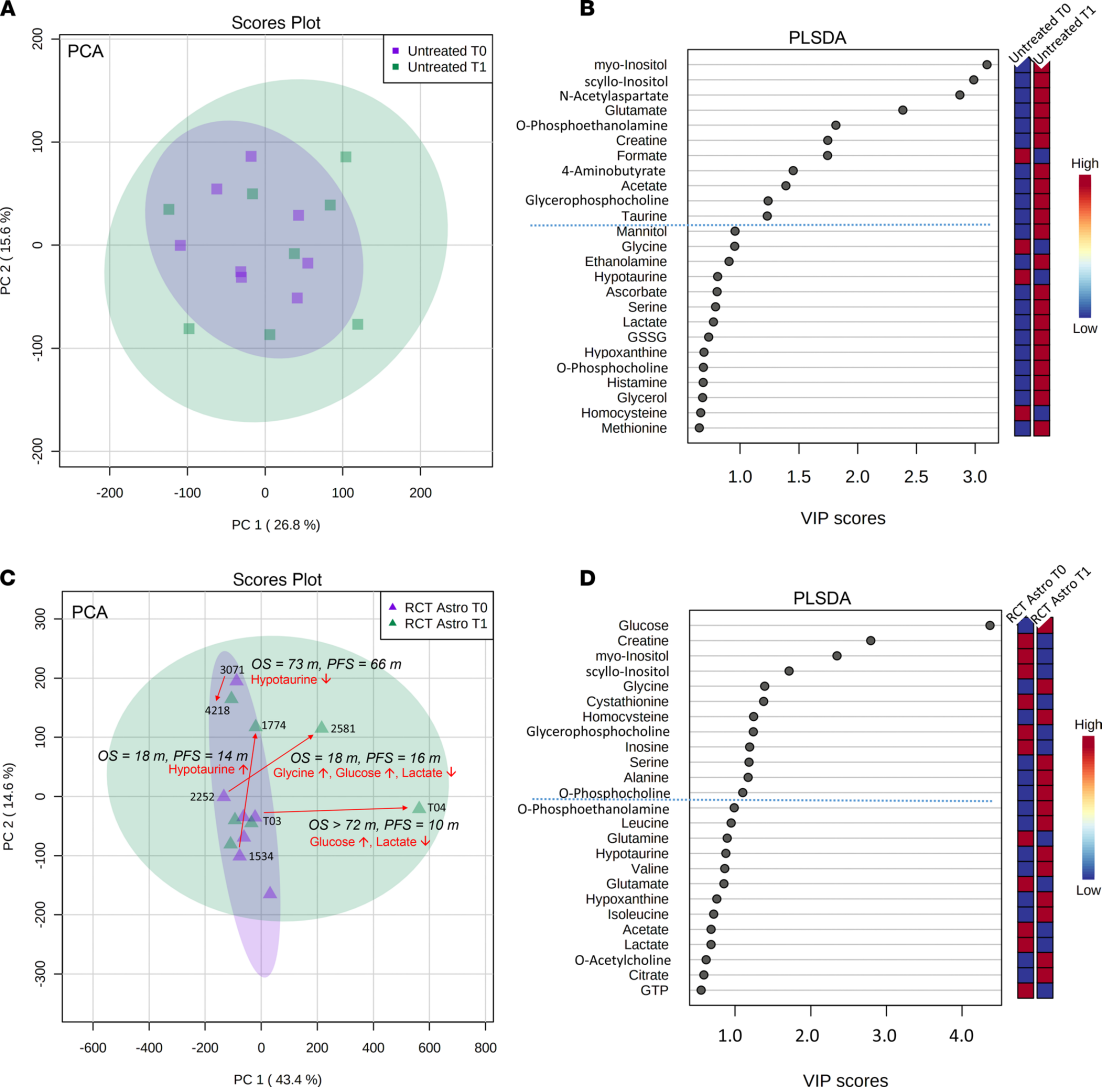
Longitudinal investigation cohort comparisons VIII and IX. (**A**) Multivariate principal component analysis (PCA) of untreated (*n* = 8) patients (comparison VIII) shows no general separation of samples from first (T0) and second (T1) surgery time point. (**B**) The partial least squares–discriminant analysis (PLS-DA) identifies 11 metabolites (on top of dashed blue line) with variable importance in projection (VIP) scores > 1 that contribute significantly to the PLS-DA model (blue box, low metabolite concentration in the respective group; red, high concentration). (**C**) By contrast, PCA of treated (*n* = 7) astrocytoma (RCT Astro) patients (comparison IX) shows 5 samples (samples 4218, 3071, 1774, 2581, T04) outside the main cluster. From those, we identified 4 sample pairs with specific metabolite changes between T0 and T1; while patient 3071/4218 showed decreased hypotaurine levels and higher OS (73 months) and PFS (66 months), patient 1534/1774 showed the opposite, with an increase in hypotaurine and a very short OS (18 months) and PFS (14 months). The patient sample pairs 2252/2581 (OS 18 months, PFS 16 months) and patient T03/T04 (OS > 72 months, PFS 10 months) showed a strong increase in glucose with decreased lactate. However, a reduced OS in patient 2252/2581 was additionally associated with increased glycine levels between the 2 resections. (**D**) The PLS-DA identified 12 further metabolites (on top of dashed blue line) with variable importance in projection (VIP) scores > 1 that contribute significantly to the PLS-DA model. m, months.

**Figure 8:**
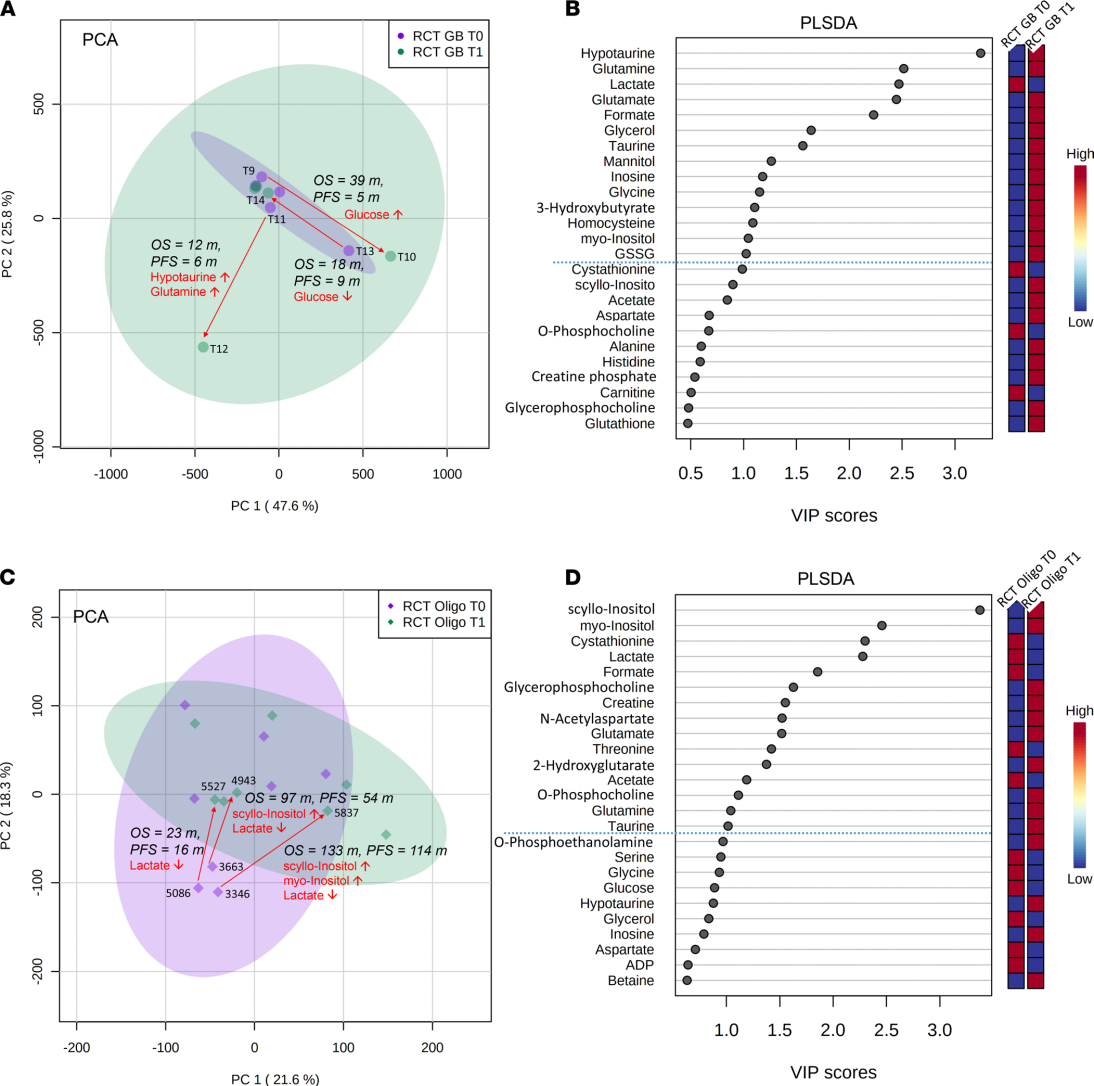
Longitudinal investigation cohort comparisons X and XI. (**A**) Multivariate principal component analysis (PCA) of treated glioblastoma (RCT GB) (*n* = 5) patients (comparison X) shows 3 samples outside the main cluster. The corresponding pairs are T11/T12 with the shortest OS (12 months) and PFS (6 months) in this comparison (associated with an increase of hypotaurine and glutamine), T13/T14 (PFS 9 months, OS 18 months) with a decrease of glucose, and T09/T10 with the longest OS (39 months) and PFS (5 months) and glucose increase. (**B**) The partial least squares–discriminant analysis (PLS-DA) identifies 14 metabolites (on top of dashed blue line) with variable importance in projection (VIP) scores > 1 that contribute significantly to the PLS-DA model (blue box, low metabolite concentration in the respective group; red, high concentration). (**C**) Next, PCA of treated (*n* = 7) oligodendroglioma (RCT Oligo) patients (comparison XI) shows 3 samples (5086, 3663, 3346) outside the main cluster. All 3 corresponding patient pairs showed a decrease of lactate first (T0) to second tissue resection time (T1); however, additional increase of scyllo- and myo-inositol extended overall and PFS in 3663/4943 (OS 97 months, PFS 54 months) and 3346/5837 (OS 133 months, PFS 114 months), while pair 5086/5527 showed the shortest OS (23 months) and PFS (16 months). (**D**) The PLS-DA identified 15 further metabolites (on top of dashed blue line) with variable importance in projection (VIP) scores > 1 that contribute significantly to the PLS-DA model. m, months.

We performed principal component analysis (PCA) and PLS-DA analysis for comparisons in the Longitudinal investigation cohort (Figure 1B and Figures 7 and 8) and correlated them with clinical data (Supplemental Table 2).

Comparison VIII (Figure 1B) with untreated tissue pairs did not show any separation or clustering in the corresponding scores. We did not detect any patient-specific alterations — i.e., longitudinal alterations in tumor tissues from the same patient. In addition, we investigated general tissue metabolite differences in comparison VIII (Figure 7, A and B) between both time points — i.e., a pooled comparison of all newly diagnosed tissues and all tissues at first progression without any treatment between both resections. We identified in the PLS-DA 11 differentiating metabolites with VIP scores > 1.0, including myo-inositol, scyllo-inositol, N-acetylaspartate, glutamate, and O-phosphoethanolamine (Figure 7, A and B).

Comparison IX (Figure 1B) (treated astrocytoma WHO grades II/III) identified 3 major clusters in the PCA scores and a total of 12 metabolites, including glucose, creatine, myo-inositol, scyllo-inositol, and glycine, with VIP scores > 1.0 in the PLS-DA (Figure 7, C and D). Major differences in the PCA were mainly shaped by these sample pairs: 3071/4218 (PFS, 66 months; OS, 73 months), 1534/1774 (PFS, 14 months; OS, 18 months), 2252/2581 (PFS, 16 months; OS, 18 months), and patient T03/T04 (PFS, 10 months; OS > 72 months), driven by lactate, hypotaurine, glycine, and glucose.

In comparison X (Figure 1B), we investigated tissue pairs of treated glioblastoma (Figure 8, A and B). We identified 2 major clusters in the PCA associated with the following samples: T11/T12 (PFS, 6 months; OS, 12 months), T09/T10 (PFS, 5 months; OS, 39 months), and T13/T14 (PFS, 9 months; OS, 18 months). Major patient-specific differences were identified for glucose, glutamine, and hypotaurine. General tissue metabolite differences included 14 metabolites with VIP scores > 1.0 were identified including hypotaurine, glutamine, lactate, glutamate, and formate (Figure 8, A and B).

By comparison XI (Figure 1B), comparing treated oligodendroglioma tissues (Figure 8, C and D), we identified 1 major cluster in the PCA with 3 sample pairs: 5086/5527 (PFS, 16 months; OS, 23 months), 3663/4943 (PFS, 54 months; OS, 97 months); 3346/5837 (PFS, 114 months; OS, 133 months). All 3 patient-specific sample pairs showed a decrease in lactate after treatment. Furthermore, we observed 15 general tissue metabolite differences with VIP scores > 1.0 in the PLS-DA, including scyllo-inositol, myo-inositol, cystathionine, lactate, and formate (Figure 8D).

### Tissue metabolites correlate with clinical outcome parameters.

Finally, we examined the relationship of tissue metabolites at initial diagnosis with progression-free survival (PFS) and overall survival (OS). First, we determined the relevant threshold for each metabolite (Supplemental Figure 4). For correlations with PFS, we identified 13 metabolites: PFS increased, with increasing concentrations of 2-HG, creatine phosphate, myo-inositol, and scyllo-inositol, while PFS decreased, with increasing concentrations of alanine, homocysteine, isoleucine, leucine, mannitol, o-acetylcholine, serine, threonine, and sn-glycero-phosphocholine (Supplemental Figure 4, A and B).

**Figure 9:**
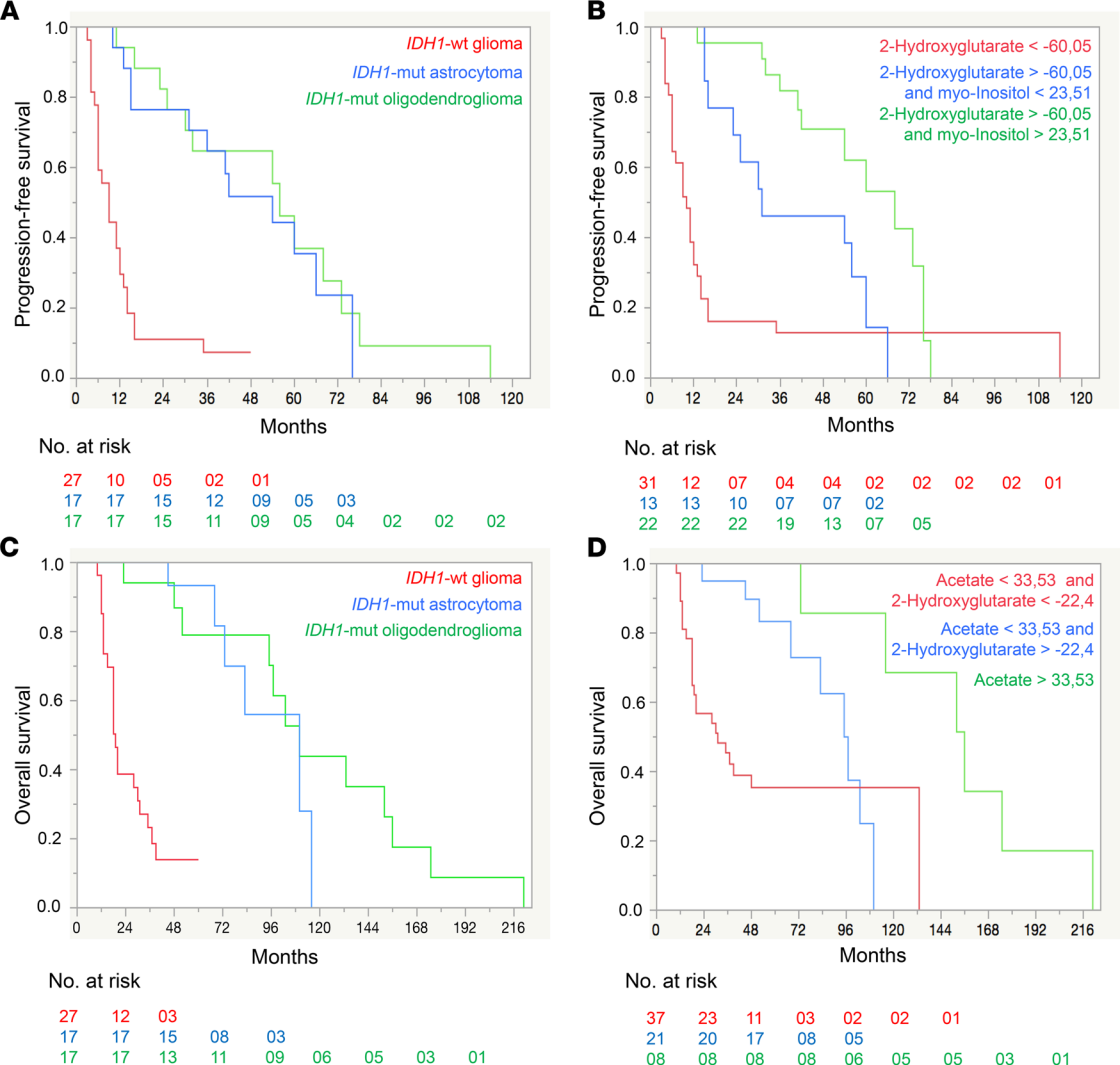
Tissue metabolite Kaplan-Meier survival curves of progression-free survival and overall survival. (**A**–**D**) Kaplan-Meier survival curves of the PFS (**A** and **B**) and OS (**C** and **D**) for IDH1-WT (*n* = 27), IDH1-mut astrocytoma (*n* = 17), and IDH1-mut oligodendroglioma (*n* = 17) and for groups defined by selected metabolites and their patient cohort-specific thresholds. The groups with the worst PFS and OS are shown in red, with intermediate PFS and OS in blue, and with the best PFS and OS in green. Metabolite concentrations (mM) were normalized and Pareto scaled for dilution effects. Log-rank test *P* < 0.0001 for all the 4 curves.

For correlations with OS, we discovered 15 metabolites: OS increased with increasing concentrations of 2-HG, acetate, formate, myo-inositol, and sn-glycero-phosphocholine. Furthermore, OS decreased with increasing concentrations of alanine, glutamate, glycine, homocysteine, isoleucine, leucine, mannitol, phenylalanine, threonine, and tyrosine (Supplemental Figure 4, C and D). The Kaplan-Meier curves indicated that prognostic values of distinct tissue metabolites (2-HG and myo-inositol for PFS, and 2-HG and acetate for OS groups 2 and 3) were clinically more favorable than the prognostic values of *IDH1* mutation or histology (Figure 9).

## Discussion

*IDH1* mutations occur in a variety of cancers, and further insight into their effects on cellular processes — including metabolism in particular cancer entities — is an important area of development. These insights will improve an understanding of context-dependent alterations, which is crucial for successful clinical translation. We performed a comprehensive tissue metabolomics and pathway analysis approach (supported by RNA-Seq) in a large cohort of diffuse glioma patients and a high number of samples. We identified distinct metabolites and metabolic pathways that were modulated by *IDH1* mutation status, histology, and tumor therapies. The correlation of metabolite with gene expression data allowed us to have a complementary look at the functional tumor biology level by an independent method (e.g., increased 2-HG levels matched with *L2HGDH* upregulation in *IDH1-*mut; ref. 15). The alterations of sn-glycero-3-phosphocholine in *IDH1*-mut (increased level) and *IDH1*-WT (decreased level) correlated well with alterations of *LYPLA1* expression (Supplemental Figure 1). Low expression of *LYPLA1* led to the accumulation of sn-glycero-3-phosphocholine (16), supporting our metabolite findings. Of note, many metabolites are substrates for more than 1 enzyme; thus, single comparisons of enzymatic activity and metabolite levels do not necessarily correlate — e.g., in the case of glutamate where we identified in the *IDH1-WT* glioblastoma group a total of 3 upregulated (*OPLAH, EARS2, GOT1*) and 2 downregulated (*GLUD1, GCLC*) enzymes (Figure 4C).

Comparisons I–III illustrate potentially novel metabolite profiles modulated by *IDH1* mutation that might further explain different clinical features and prognoses of glioma subtypes. For example, we identified increased levels of sarcosine, o-acetylcholine, and glutamate in *IDH1*-WT glioma compared with *IDH1*-mut glioma (Tables 1 and 2). Recent studies indicate that these neurotransmitters have tumor-promoting features; thus, this might help to further explain the unfavorable prognosis of *IDH1*-WT compared with *IDH1*-mut diffuse glioma (17–20).

Increased hypotaurine level was another metabolic feature of *IDH1*-WT diffuse glioma (Tables 1 and 2). Recent preclinical studies suggest that hypotaurine activates hypoxia-related signaling and mediates tumor progression in glioma (21). Of note, correlations of *ADO* gene expression and hypotaurine metabolite levels did not correlate in *IDH1*-WT glioblastoma (Figure 4C). We detected higher hypotaurine levels but reduced *ADO* gene expression in *IDH1*-WT glioblastoma. Two further enzymes that could be connected to changing hypotaurine levels were either not part of our RNA data set (glutamate decarboxylase like 1 [*GADL1*]) or only slightly increased in the *IDH1*-mut but not in *IDH1*-WT glioblastoma group (cysteine sulfinic acid decarboxylase [*CSAD*]). Thus, we cannot fully explain the lack of correlation between *ADO/GADL1/CSAD* gene expression and hypotaurine levels.

Another finding was the detection of higher cystathionine levels in *IDH1*-mut astrocytoma (Figure 3C). This metabolite was recently reported as a magnetic resonance spectroscopy (MRS) marker for the noninvasive detection of 1p/19q codeleted gliomas (22) — e.g., oligodendroglioma (which, per definition, harbor *IDH1* mutations). The identification of cystathionine in *IDH1*-mut astrocytoma in our study might indicate that this MRS parameter reflects the presence of *IDH1* mutation. The corresponding *CBS* gene expression levels were not statistically significantly altered in our study, probably due to the limited sample size.

The comparison III of *IDH1*-mut versus *IDH1*-WT glioblastoma (Figure 4C) revealed several metabolite differences. Increased glutamate tissue levels in *IDH1*-WT glioblastoma correlated with several gene expression changes (Figure 4C), indicating different roles of glutamate in multiple metabolic pathways. *GLUD* leads to glutamate turnover and is involved in metabolic pathways (nitrogen, alanine, aspartate and glutamate metabolism, arginine biosynthesis) (23). Low *GLUD1* levels in *IDH1*-WT glioblastoma, thus, correlate well with increased glutamate tissue levels. Glutamate has tumor-promoting features in glioma (20). Furthermore, citrate and corresponding *ACLY* gene expression levels were increased. *ACLY* is involved in oxaloacetate, acetyl-CoA and citrate turnover and promotes cancer cell proliferation and progression (24, 25). These metabolic differences between *IDH1*-WT (increased glutamate, citrate; Figure 4C) and *IDH1*-mut glioblastoma (decreased glutamate, citrate; Figure 4C) reflect different clinical outcome of these entities. Another finding included decreased levels of butyrate, a neuroprotective gut metabolite (26), in both *IDH1*-mut and *IDH1*-WT glioblastoma (Tables 1 and 2), suggesting further investigations on the role of microbial metabolites.

We then investigated histology-dependent comparisons IV–VII in the *IDH1* mutation status cohort (Figures 5 and 6 and Supplemental Figure 2 and 3). As outlined, the diagnoses of all different glioma entities in our cohorts from Tübingen and Cologne were based on the WHO classification 2016 (1) and the following parameter for molecular diagnostics: *IDH1*, ATRX, 1p/19q codeletion, and *MGMT*. For example, oligodendrogliomas were only diagnosed based on the presence of *IDH1* mutation and 1p/19q codeletion. Of note, the upcoming new WHO classification will introduce further entities and refine existing diagnoses (27, 28). This is particularly true for astrocytic tumors, e.g., the *IDH1*-mut glioblastoma (as diagnosed in our cohort based on the current WHO classification) will be replaced by the entity *IDH1* mutated astrocytoma grade 4. Since our metabolite comparisons mainly focus on the ongoing parameters in diffuse glioma such as *IDH1* mutation and histomorphology, the data portrayed here will still be of a high relevance and importance even after upcoming classification changes by WHO.

The Longitudinal investigation cohort comparisons VIII–XI (Figure 1B, Figures 7 and 8, and Supplemental Table 2) investigated metabolic alterations during tumor progression either with (treated) or without treatment (untreated) between 2 resections. To overcome sample size limitations in the treated category, we pooled all tumor therapies. We did not detect patient-specific significant alterations in untreated longitudinal comparisons (Figure 7A). In comparisons IX–XI, the PCA (Figures 7 and 8) revealed several outliers from the main clusters, with glucose, lactate, glycine, scyllo/myo-inositol, hypotaurine, and glutamine being the major drivers of separation. For 1 patient with glioblastoma (T10/T11) (Figure 8A), we found a significant increase of hypotaurine and glutamine, both of which have been previously reported as tumor-promoting metabolites (21, 29). Of note, PFS and OS of this patient were lower compared with other patients in this comparison. Finally, we matched pairs of oligodendroglioma (comparison XI; Figure 8C and Supplemental Table 2); in the PCA, we identified 1 cluster of 3 patients driven by lactate, which decreased over time (Figure 8C), indicating a reduced Warburg effect. Clinical outcome in 2 of these patients who had an additional increase in scyllo- and myo-inositol concentrations showed increased PFS and OS compared with other patients in this comparison. Even with rather small sample sizes in the comparisons of the Longitudinal investigation cohort, we provide candidate metabolites for the design of prospective studies. Particularly, we provide an evaluation on longitudinal levels of hypotaurine, glutamine, and lactate, and their correlations with clinical outcome might identify novel surrogate markers. However, further investigations in larger prospective cohorts will be necessary to dissect whether these longitudinal patient-specific metabolite alterations are consequences of their treatment rather than tumor progression per se. Our present study suggests candidate tissue metabolites for such a prospective evaluation.

Finally, we investigated the potential utility of tissue metabolites as prognostic markers by correlating metabolite levels at initial diagnosis with PFS and OS (Figure 9 and Supplemental Figure 4). A recent study (30) identified 9 metabolites that correlated significantly with OS but did not investigate any correlations of metabolites with PFS. We detected 13 metabolites that predicted PFS (Supplemental Figure 4A) and 15 metabolites that correlated with OS (Supplemental Figure 4C). We confirmed previous correlations with OS for myo-inositol, alanine, sn-glycero-3-phosphocholine, glycine, and glutamate. However, we did not detect any associations with OS for choline, creatine, and phosphocholine. In our study, increased acetate levels correlated positively with OS. In addition to these metabolites, we discovered 9 potentially novel metabolites that should be further evaluated as prognostic markers for OS (Figure 9 and Supplemental Figure 4). Our data suggest that systematic prospective assessments of tissue metabolic profiling and their correlation with PFS and OS in prospective large data sets are warranted. They might help to further augment and refine existing integrated diagnoses and prognostic stratification in given glioma subtypes.

Taken together, this in-depth analysis of tissue metabolomics and altered metabolic pathways, supported by RNA-Seq, in diffuse glioma provides insights into metabolite alterations in distinct and clinically relevant subgroups of diffuse glioma. It can serve as a starting point for multiple further experimental investigations and prospective clinical studies to further understand exact underlying molecular mechanisms and modes of action.

## Methods

### Patients and samples.

This study was approved by the institutional ethics committee. We retrospectively identified 73 patients from the University Hospital Tübingen and the University Hospital Cologne, with diffuse glioma WHO grades II–IV with archival high-quality tissue samples, molecular routine diagnostics for IDH1 status, 1p/19q, ATRX, and MGMT gene methylation according to the WHO classification 2016 (1). Furthermore, the diagnosis of oligodendroglioma always included the presence of *IDH1* mutation and 1p/19q codeletion. Among these patients, 28 of 73 patients had more than 1 resection, leading to a total of 101 samples. Patients had been treated at the University Hospital Tübingen between 2014 and 2017 (*n* = 53) and at the University Hospital Cologne (*n* = 20) between 1999 and 2014. All demographic and clinical characteristics were collected from the electronic clinical database. We defined 2 cohorts (Figure 1): The *IDH1* mutation status cohort and the Longitudinal investigation cohort and performed distinct comparisons of tissue metabolites (comparisons I–XI; Figure 1).

### Metabolite and RNA extraction from archival biological samples.

Archival tumor samples were available after tissue acquisition according to a standardized routine biobanking procedures. In brief, after removal of tumor tissue from the resection cavity in the operating room, samples were immediately frozen in liquid nitrogen, the tumor content was assessed on a cryo-slide by a neuropathologist, and only tissue samples with sufficient tumor content (>70%–80%) were stored as biological samples in the biobank at –80°C. Even though samples were immediately frozen after tissue removal, it is known that neurosurgical tissue collection might also involve occlusion of blood vessel supply and, thus, also lead to metabolic changes in the context of surgical procedures. We consider this as a natural limitation of investigations of tissue metabolomics. Deep-frozen biopsy sample (50–100 mg) were cryogenically pulverized (cryoPREP, Covaris Inc.) and dried in a lyophilisator overnight (Alpha 2–4, Martin Christ). A target mass of 5 mg dried sample was used for metabolite extraction by focused ultrasonication, and 3 mg of dry weight was used for RNA isolation.

### NMR-based metabolomics.

A target mass of 5 mg lyophilized glioma powder was transferred into 2 mL adaptive focus acoustics (AFA) glass tubes (milliTUBE, Covaris Inc.) and mixed with 400 μL of ultrapure methanol and 800 μL of MTBE (solvent grade). The tissue was then homogenized in a degassed water bath at 7°C with a focused ultrasonicator (E220evolution, Covaris Inc.). Two consecutive sonication programs with vertical sample movement each lasting 5 minutes were used for a maximum yield of homogenization and metabolite extraction. Afterward, the metabolite suspension was separated into a polar and lipid phase by adding 600 μL of ultrapure water. Then, the glass tubes were centrifuged for 5 minutes at 5000*g* at 4°C. The polar phase was evaporated to dryness overnight with a vacuum concentrator (Concentrator 5310, Eppendorf). Dried pellets were resuspended with deuterated phosphate buffer (200 mM K_2_HPO_4_, 200 μM NaN_3_, pH 7.4) containing 1 mM of the internal standard TSP (trimethylsilylpropanoic acid). To obtain a maximum dissolution, the plastic tubes were thoroughly vortexed and then centrifuged 5 minutes at 14,000*g* at 4°C. The clear supernatants were transferred into 5 mm and 1.7 mm NMR tubes and a 96-well rack placed into the cooled (4°C) NMR autosampler. Spectra were recorded on a 600 MHz ultrashielded NMR spectrometer (Avance III HD, Bruker BioSpin) equipped with a 5 mm (patients with 1 sampling time point) and 1.7 mm (patients with 2 sampling time points) triple-resonance (^1^H, ^13^C, ^15^N/^31^P) room temperature probe. For optimum water suppression and shim adjustment, a quick simple proton zero go (ZG) experiment was performed followed by a 2-hour lasting Carr-Purcell Meiboom-Gill (CPMG) experiment in order to suppress residual background signals from macromolecules (time domain, 64,000 points; sweep width, 20 ppm; 1024 scans). The recorded free induction decays (FIDs) were fourier transformed and were spectra properly phase corrected and baseline corrected (Topspin 3.6.1, Bruker BioSpin). Metabolite annotation and quantification was performed with Chenomx NMR Suite 8.5 (Chenomx Inc.).

### RNA-Seq.

RNA-Seq was performed at the Next Generation Sequencing Competence Center Tübingen (NCCT) on an Illumina NovaSeq6000 instrument (Illumina) with a sequencing depth of approximately 25 million clusters per sample.

RNA was purified from lyophilized tissue using the QIAsymphony RNA kit (Qiagen). Tissue was thawed at room temperate, and 800 μL of Buffer RTL Plus was added to the tissue. Homogenization was performed twice for 2 minutes at 25 Hz using the TissueLyser II (Qiagen). Resulting homogenates were centrifuged for 3 minutes at 14,000*g* and 4°C at maximum speed in a microcentrifuge, and 400 μL of the resulting lysate was loaded on the QIAsymphony SP (Qiagen) using the RNA CT 400 protocol. At the end of the protocol, the RNA was eluted in 35 μL of RNase-free water.

For library preparation, mRNA fraction was enriched using polyA capture from 200 ng of total RNA using the NEBNext Poly(A) mRNA Magnetic Isolation Module (NEB) with the liquid handler Biomek i7 (Beckman). Next, mRNA libraries were prepared using the NEB Next Ultra II Directional RNA Library Prep Kit for Illumina (NEB) according to the manufacturer’s instructions. Library molarity was determined by measuring the library size (approximately 400 bp) using the Fragment Analyzer with the High NGS Fragment (1–6000 bp assay; Agilent) and measuring the library concentration (approximately 5 ng/μL) using the Infinite 200Pro (Tecan) and the Quant-iT HS Assay Kit (Thermo Fisher Scientific). The libraries were denatured, diluted to 270 pM, and sequenced as paired-end 100 bp reads on an Illumina NovaSeq6000 (Illumina) with a sequencing depth of approximately 25 million clusters per sample.

Read quality of RNA-Seq data in FASTQ files was assessed using ngs-bits (v.2020_06) to identify sequencing cycles with low average quality, adaptor contamination, or repetitive sequences from PCR amplification. Reads were aligned using STAR v2.7.3a to the GRCh37, and alignment quality was analyzed using ngs-bits and visually inspected in the Integrative Genome Viewer (v2.7.2). Normalized read counts for all genes were obtained using Subread (v2.0.0).

### Selection and presentation of significant metabolites by NMR spectroscopy and RNA-Seq in comparisons I–XI.

Using spectral analysis of ^1^H-NMR data, we annotated and quantified a total of 66 metabolites, and 58 showed significant changes as defined by *P* < 0.05 (we additionally set a fold change threshold of > 1.2 to avoid the display of variations close to background) in at least 1 of the comparisons I–XI (Figure 1, A and B, and Tables 1 and 2). We also displayed metabolites that we detected with a lower threshold for significance — i.e., with *P* < 0.1 (Tables 1 and 2) for a total of 81 metabolites. This extended set is important because the sample sizes per comparison might have been too low to detect higher significance levels in univariate testing. In the *IDH1* mutation status cohort (Figure 1A and Tables 1 and 2, comparisons I–III), we found a total of 39 significant metabolites and another 25 metabolites with *P* < 0.1. Additionally, within the *IDH1* mutation status cohort, we investigated metabolic tissue profiles with identical *IDH1* mutation status and different histology, and we identified a total of 44 significant and additional 22 metabolites with *P* < 0.1 (Figure 1A and Tables 1 and 2, comparisons IV–VII).

Finally, in the Longitudinal investigation cohort, we detected a total of 5 significant and 14 more metabolites with *P* < 0.1 (Figure 1B and Tables 1 and 2, comparisons VIII–XI).

We used the gene expression data from RNA-Seq for a cross-correlation of metabolite/gene expression changes in the *IDH1* mutation status cohort where metabolites of *IDH1*-mut were compared against *IDH1*-WT (comparisons I–II).

### Data availability.

Metabolomics data have been deposited to the EMBL-EBI MetaboLights database (DOI: 10.1093/nar/gkz1019, PMID:31691833) with the identifier MTBLS3873. The complete data set can be accessed at https://www.ebi.ac.uk/metabolights/MTBLS3873 RNA-Seq data have been deposited in NCBI’s Gene Expression Omnibus and are accessible through accession no. GSE190504 (https://www.ncbi.nlm.nih.gov/geo/query/acc.cgi?acc=GSE190504).

### Statistics.

All statistical analyses were performed with the MetaboAnalyst 4.0 toolbox with a threshold of maximum 60% missing values and with JMP 14.2.0 (SAS Institute Inc.; https://www.jmp.com/en_us/home.html). In order to account for dilution and different tissue weights/cell numbers, we normalized data using the probabilistic quotient normalization (PQN) approach (31). Furthermore, for making metabolites of high magnitude changes comparable, the pareto scaling method was applied (mean-centered and divided by the square root of the SD of each variable), which can result in negative values (32). The preprocessed data were analyzed by univariate classical 2-tailed Student’s *t* test (*P* < 0.05 and FDR < 0.1 were considered significant), multivariate PCA including combined scores and loadings plot and multivariate PLS-DA including VIP scores where metabolites with VIP scores > 1 represent a significant contribution to the PLS-DA model (VIP scores are calculated as a weighted sum of the squared correlations between the PLS-DA components and the original variable).

ROC analyses were performed to identify robust biomarkers that can distinguish between the effects of *IDH1* mutation and histology entity. Top AUC (area under the ROC curve) metabolites were screened for related gene expression changes in the RNA data set for comparisons I–III.

Pathway analysis was performed with the quantitative enrichment analysis module of the MetaboAnalyst 4.0 toolbox. Metabolite abundance changes from the comparisons I–III and IV–VII (Figure 1A) were matched with the Kyoto Encyclopedia of Genes and Genomes (KEGG) database with 84 metabolite sets based on human pathways.

For correlations of metabolites with PFS and OS, we performed univariate regressions for each metabolite. All metabolites that showed significant correlations (likelihood-ratio test, *P* < 0.05) for PFS and OS were considered for further analysis. One-stage classification and regression tree (CART) analyses identified the optimal thresholds for each metabolite to dichotomize patients into good and poor survival groups. Kaplan-Meier analyses estimated survival for all subgroups of patients stratified by the identified metabolites and their thresholds. Survival times are presented as median survival with 95% CI. Furthermore, we performed exemplary 2-stage CART analyses to identify the 2 most prognostic metabolites and their thresholds that correlated with PFS and OS to stratify patients into 3 prognostic groups: good, intermediate, and poor survival (groups 1–3). In addition, we also used 2-stage CART analyses to stratify patients into prognostic groups according to defined criteria — i.e., *IDH1* mutation, histologic diagnosis, and WHO grade. Correlations with survival were determined by Kaplan-Meier analyses and Kaplan-Meier curves to compare both models for stratifying patients into prognostic groups of PFS and OS.

### Study approval.

The study was approved by the ethical board of the University Hospital Tübingen (reference no. 484/2016BO1; December 7, 2016, and August 18, 2021).

## Author contributions

Study concept and experimental design were contributed by GT, CT, and MS. Implementation of experimental design and data acquisition were contributed by CT, IM, LZ, MS, B. Bayer, SJG, and GT. Statistical analysis was contributed by CT, LZ, and MS. Data analysis and interpretation were contributed by CT, LZ, IM, MS, B. Bender, B. Bayer, MT, UE, SJG, BJP, and GT. Writing of the manuscript was contributed by CT, LZ, MS, and GT. Figures and tables were contributed by CT, LZ, and MS. All authors gave final approval of the manuscript.

## Supplementary Material

Supplemental data

## Figures and Tables

**Table 1:**
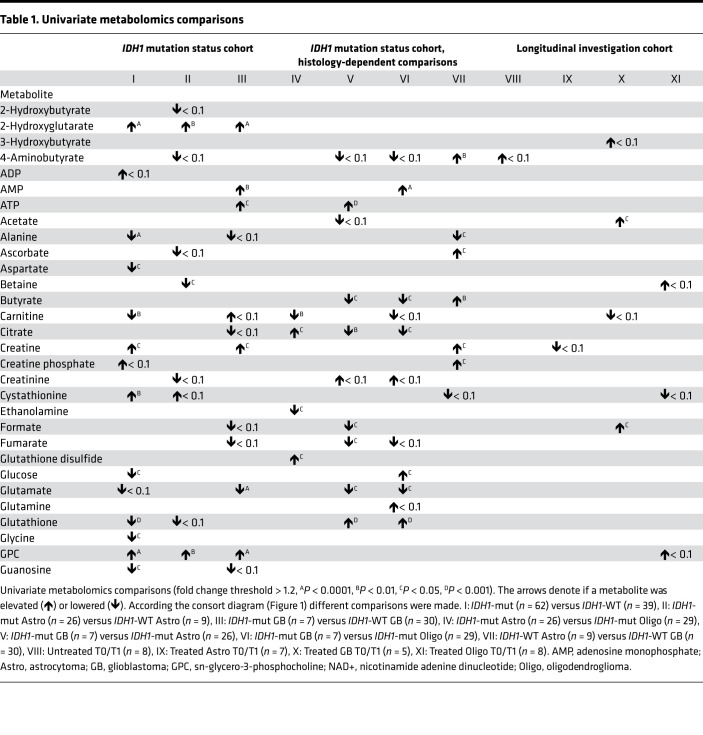
Univariate metabolomics comparisons

**Table 2:**
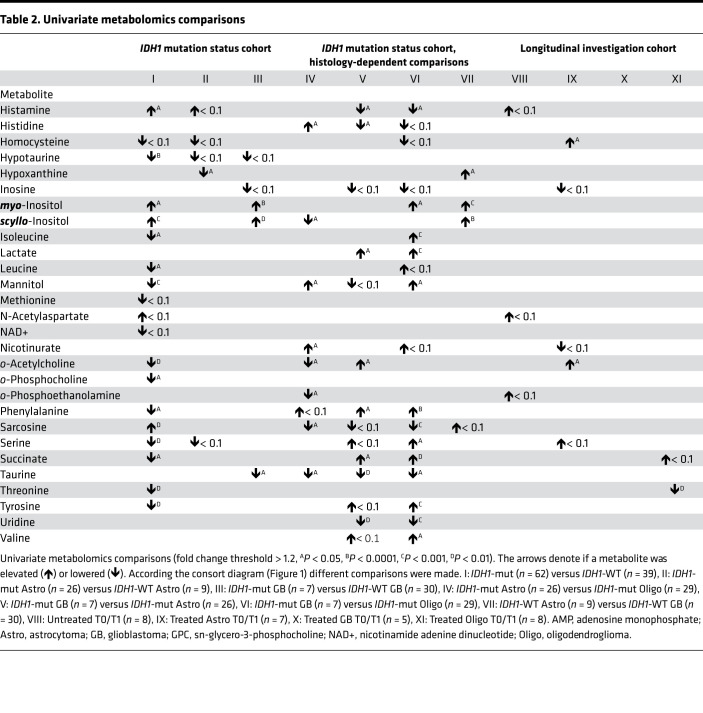
Univariate metabolomics comparisons

## Version 1 12/23/2021

In-Press Preview

## Version 2 02/08/2022

Electronic publication
