# Supplementary material for: Tissue metabolites in diffuse glioma and their modulations by IDH1 mutation, histology, and treatment
Source: JCI Insight. 2022 Feb 8;7(3):e153526. doi: 10.1172/jci.insight.153526 (PMC8855807; doi:10.1172/jci.insight.153526)
Supplement: Supplemental data [file jciinsight-7-153526-s253.pdf]

<u>Supplementary information (Trautwein *et al.*)</u>

Content

1 Supplementary figures S1-S5

2 Supplementary tables ST1-ST2

1 Supplementary Figures

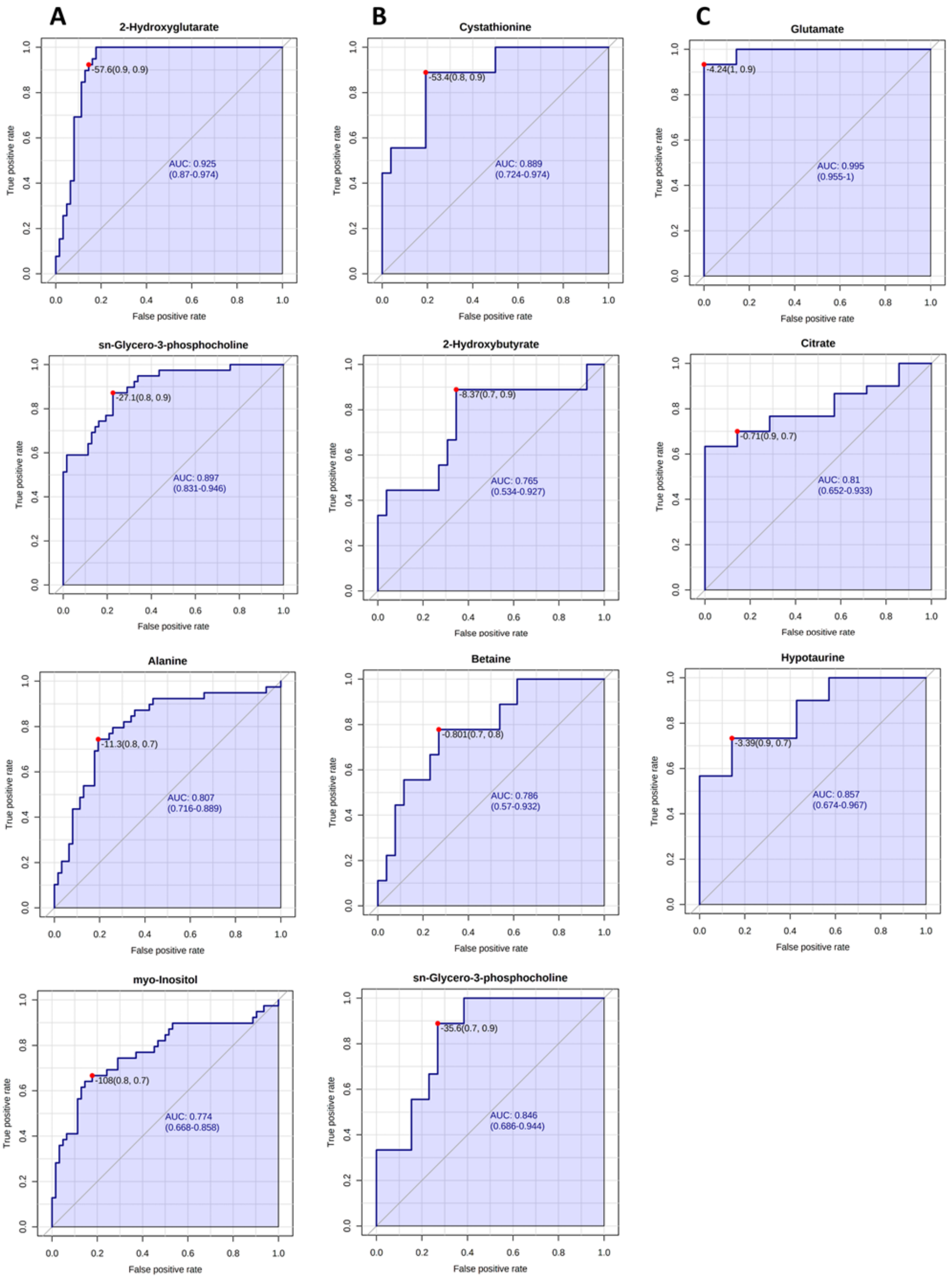

Supplementary Figure S1: Receiver operating characteristic (ROC) analysis.
Area under the curve (AUC) plots for *IDH1*-mutation (*IDH1*-mut, n = 62) vs. wildtype (*IDH1*-wt, n = 39) comparison **I** (**A**), astrocytoma (Astro) *IDH1*-mut (n = 26) vs. wt (n = 9) comparison **II** (**B**) and glioblastoma (GB) *IDH1*-mut (n = 7) vs. wt (n = 30) comparison **III** (**C**).

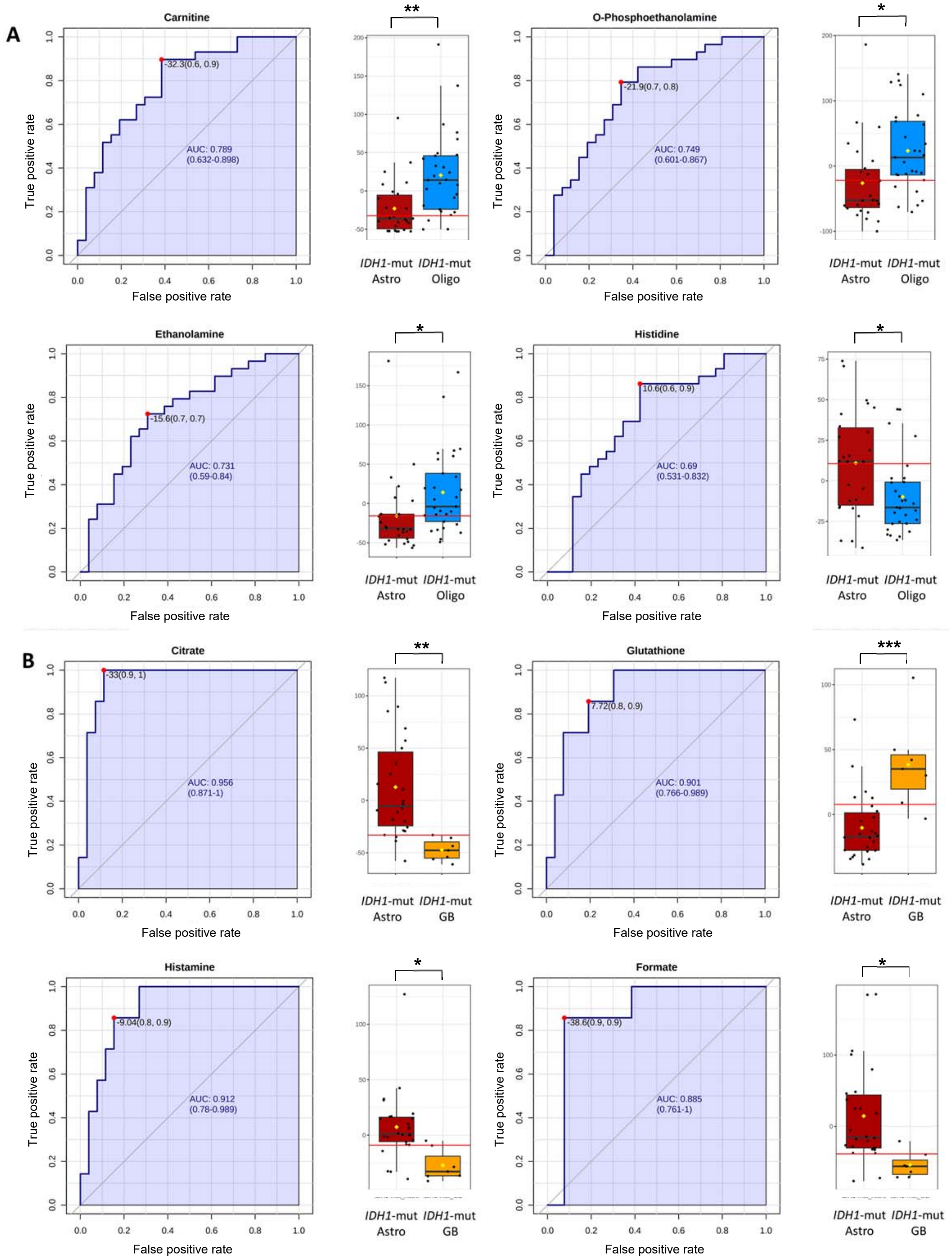

Supplementary Figure S2: Comparisons of tumor entities by receiver operating characteristic ROC analysis and significant metabolites. (**A**) Astro *IDH1*-mut (n = 26) vs. Oligo *IDH1*-mut (n = 29) comparison **IV**, and (**B**) Astro *IDH1*-mut (n = 26) vs. GB *IDH1*-mut (n = 7) comparison **V**. *p*-values determined by student's t-tests. Illustrated as box and whiskers plots showing all points with mean, min/max and outliers. Abbreviations: Astro, astrocytoma; GB, glioblastoma; Oligo, oligodendroglioma. *p*-values: *** < 0.001, ** < 0.01, * < 0.05.

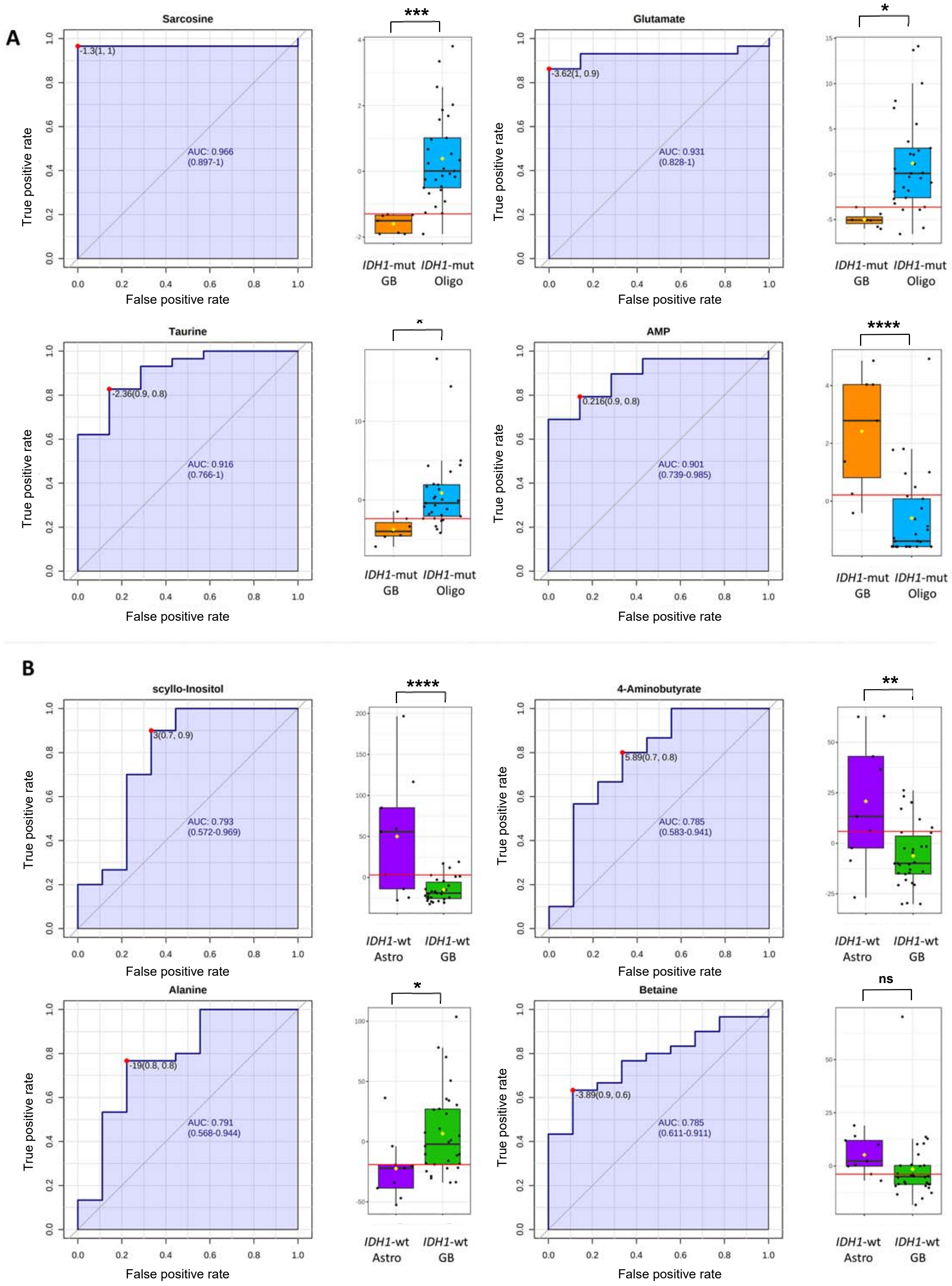

Supplementary Figure S3: Comparisons of tumor entities by receiver operating characteristic ROC analysis and significant metabolites. (**A**) GB *IDH1*-mut (n = 7) vs. Oligo *IDH1*-mut (n = 29), comparison **VI**, and (**B**) Astro *IDH1*-wt (n = 9) vs. GB *IDH1*-wt (n = 30) comparison **VII**. *p*-values determined by student's t-tests. Illustrated as box and whiskers plots showing all points with mean, min/max and outliers. Abbreviations: Astro, astrocytoma; GB, glioblastoma; Oligo, oligodendroglioma. *p*-values: **** < 0.0001, *** < 0.001, ** < 0.01, * < 0.05.

A Progression-free survival

PFS<br>Univariate	Thresholds<br>(mM)#	Median (months)<br>below	 above
2-Hydroxyglutarate	-87.44	09, CI 95% 06-12, n=23	54, CI 95% 31-60, n=43
Alanine	-43.06	56, CI 95% 31-68, n=22	14, CI 95% 10-30, n=44
Creatine phosphate	-20.52	12, CI 95% 06-23, n=24	54, CI 95% 30-66, n=42
Homocysteine	40.40	36, CI 95% 16-56, n=58	11, CI 95% 03-14, n=08
Isoleucine	-3.58	41, CI 95% 23-60, n=45	12, CI 95% 06-16, n=21
Leucine	56.06	36, CI 95% 16-56, n=56	08, CI 95% 04-12, n=10
Mannitol	-48.33	54, CI 95% 14-66, n=17	16, CI 95% 12-36, n=49
myo-Inositol	-23.51	14, CI 95% 09-23, n=34	60, CI 95% 32-76, n=32
o-Acetylcholine	18.75	36, CI 95% 16-56, n=53	11, CI 95% 04-16, n=13
scyllo-Inositol	-80.09	11, CI 95% 06-14, n=20	54, CI 95% 31-66, n=46
Serine	-61.86	56, CI 95% 14-76, n=11	16, CI 95% 12-36, n=55
Threonine	18.95	41, CI 95% 16-60, n=48	12, CI 95% 06-16, n=18
sn-Glycero-3-phosph.	-22.31	42, CI 95% 25-60, n=37	12, CI 95% 09-30, n=29

B

Bivariate pathology (*IDH1*-status, pathology)		
Group 1 *IDH1*-wt	09, CI 95% 06-12,	n=27
Group 2 *IDH1*-mut, astrocytoma	54, CI 95% 50-76,	n=17
Group 3 *IDH1*-mut, oligodendroglioma	56, CI 95% 25-73,	n=17

IDH1-mut glioblastoma (n=5) were excluded, because 4 were censored.

Bivariate metabolites (2-Hydroxyglutarate -60,05 and *myo*-Inositol -23,51)		
Group 1 (2-Hydroxyglutarate < -60,05)	10, CI 95% 06-12,	n=31
Group 2 (2-Hydroxyglutarate > -60,05 and < *myo*-Inositol 23,51)	31, CI 95% 16-60,	n=13
Group 3 (2-Hydroxyglutarate > -60,05 and > *myo*-Inositol 23,51)	68, CI 95% 42-76,	n=22

C Overall survival

OS<br>Univariate	Thresholds<br>(mM)#	Median (months)<br>below	 above
2-Hydroxyglutarate	-42.99	30, CI 95% 18-48, n=36	103, CI 95% 73-152, n=30
Acetate	33.53	68, CI 95% 35-97, n=58	156, CI 95% 72-221, n=8
Alanine	-39.59	110, CI 95% 73-152, n=28	35, CI 95% 20-95, n=38
Formate	40.51	73, CI 95% 37-103, n=58	152, CI 95% 23-175, n=8
Glutamate	-32.71	97, CI 95% 68-152, n=33	37, CI 95% 19-103, n=33
Glycine	-44.75	110, CI 95% 68-156, n=29	45, CI 95% 20-83, n=37
Homocysteine	-15.84	133, CI 95% 35-133, n=28	73, CI 95% 30-103, n=38
Isoleucine	-11.02	95, CI 95% 52-110, n=42	35, CI 95% 18-open, n=24
Leucine	11.74	95, CI 95% 68-110, n=49	23, CI 95% 13-open, n=17
Mannitol	10.74	97, CI 95% 52-110, n=48	20, CI 95% 13-83, n=18
myo-Inositol	-163.2	24, CI 95% 13-97, n=11	95, CI 95% 52-110, n=55
Phenylalanine	-0.08	95, CI 95% 52-110, n=42	35, CI 95% 18-open, n=24
Threonine	18.95	95, CI 95% 68-110, n=48	39, CI 95% 18-open, n=18
Tyrosine	-5.68	97, CI 95% 68-116, n=39	31, CI 95% 18-open, n=27
sn-Glycero-3-phosph.	-11.20	37, CI 95% 18-83, n=39	110, CI 95% 68-152, n=27

D

Bivariate pathology		
Group 1 *IDH1*-wt	19, CI 95% 15-30,	n=27
Group 2 *IDH1*-mut, astrocytoma	110, CI 95% 68-116,	n=1
Group 3 *IDH1*-mut, oligodendroglioma	110, CI 95% 52-156,	n=17

IDH1-mut glioblastoma (n=5) were excluded, because all were censored.

Bivariate metabolites (Acetate 33,53 and 2-Hydroxyglutarate -22,4)		
Group 1 (Acetate < 33,53 and 2-Hydroxyglutarate < -22,4)	31, CI 95% 18-133,	n=37
Group 2 (Acetate < 33,53 and 2-Hydroxyglutarate > -22,4)	95, CI 95% 68-110,	n=21
Group 3 (Acetate > 33,53)	156, CI 95% 72-221,	n=8

Supplementary Figure S4: Tissue metabolite correlations with progression free and overall survival. Overview of metabolites and their univariate thresholds determined by one-step classification and regression tree analysis for optimal dichotomization of patients for progression-free survival (PFS, **A**) and overall survival (OS, **C**); Optimal trichotomization of patients by bivariate analyses of either pathological parameters or metabolites each by two-step classification and regression tree analysis for PFS (**B**) and OS (**D**). [#]Metabolite concentrations (mM) were normalized and Pareto-scaled for dilution effects. *sn*-Glycero-3-phosph. = *sn*-Glycero-3-phosphosphocholine.

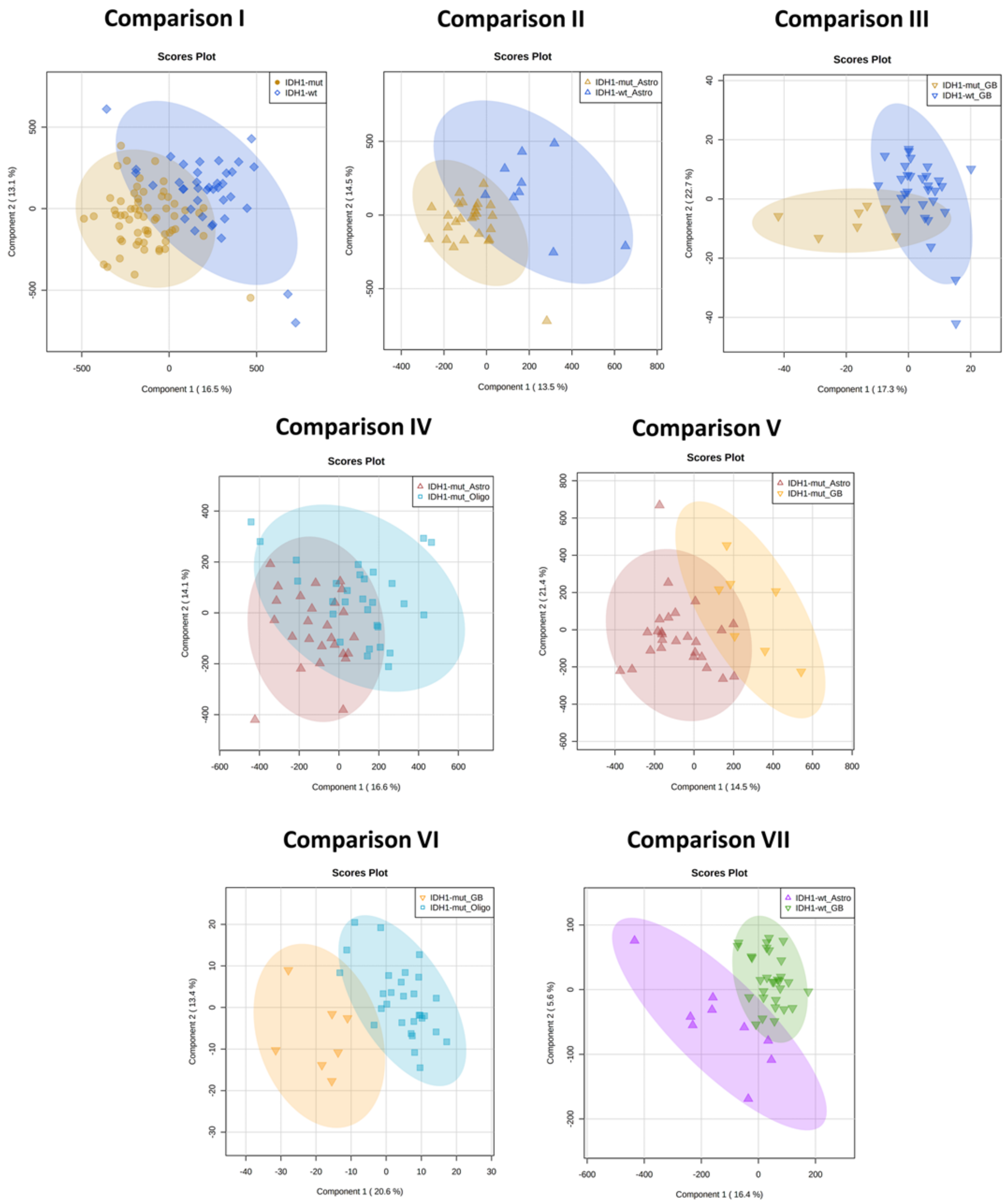

Supplementary Figure S5: Partial Least Squares Discriminant Analysis (PLSDA) scores plots from the comparisons I – VII. PLSDA scores plots were integrated in main Figures 2 – 5.

2 Supplementary tables

Comparison I: *IDH1*-mut (n = 62) vs *IDH1*-wt (n = 39)				Comparison II: *IDH1*-mut Astro (n = 26) vs *IDH1*-wt Astro (n = 9)				Comparison III: *IDH1*-mut GB (n = 7) vs *IDH1*-wt GB (n = 30)			
Biomarker	AUC	t-test	log FC2	Biomarker	AUC	t-test	log FC2	Biomarker	AUC	t-test	log FC2
**2-Hydroxyglutarate**	**0.923**	**8.95E-10**	**6.59**	**2-Hydroxyglutarate**	**0.880**	**0.003**	**3.47**	**Glutamate**	**0.990**	**7.30E-05**	**-3.55**
**sn-Glycero-3-phosphocholine**	**0.895**	**5.95E-11**	**2.96**	Cystathionine	0.876	0.052	3.72	**myo-Inositol**	**0.924**	**4.25E-06**	**-0.43**
**scyllo-Inositol**	**0.833**	**8.97E-04**	**2.96**	**sn-Glycero-3-phosphocholine**	**0.838**	**0.001**	**1.36**	**sn-Glycero-3-phosphocholine**	**0.905**	**1.32E-06**	**-0.62**
**Alanine**	**0.804**	**6.77E-07**	**0.37**	**Betaine**	**0.782**	**0.007**	**-0.88**	**scyllo-Inositol**	**0.876**	**5.57E-04**	**-1.03**
**myo-Inositol**	**0.770**	**0.020**	**2.57**	Histamine	0.774	0.074	1.35	**Hypotaurine**	**0.848**	**0.062**	**-4.80**
**Hypotaurine**	**0.758**	**7.30E-05**	**-0.34**	2-Hydroxybutyrate	0.752	0.065	0.99	**Citrate**	**0.805**	**0.061**	**-3.51**
**Sarcosine**	**0.729**	**0.002**	**3.47**	Valine	0.735	0.438	0.75	**AMP**	**0.800**	**0.012**	**2.02**
**Cystathionine**	**0.698**	**0.004**	**4.24**	**Hypoxanthine**	**0.701**	**0.021**	**-0.96**	Formate	0.786	0.081	-3.34
**Glutathione**	**0.693**	**2.72E-04**	**1.39**	ADP	0.697	0.177	2.29	**Creatine**	**0.779**	**0.008**	**-1.46**
**Mannitol**	**0.691**	**5.25E-04**	**0.78**	O-Phosphocholine	0.692	0.082	-0.59	**Alanine**	**0.776**	**0.038**	**-3.25**
**O-Acetylcholine**	**0.689**	**0.002**	**0.81**	Leucine	0.688	0.900	0.42	**Inosine**	**0.776**	**0.047**	**-3.49**
**Serine**	**0.686**	**7.96E-04**	**0.82**	**Hypotaurine**	**0.684**	**0.033**	**-0.68**	**Taurine**	**0.774**	**0.030**	**-3.46**
**O-Phosphocholine**	**0.683**	**0.011**	**0.87**	**O-Acetylcholine**	**0.679**	**0.048**	**-0.44**	Acetate	0.767	0.195	-2.98
Creatine phosphate	0.678	0.333	2.30	Glutamate	0.671	0.174	-0.29	Guanosine	0.745	0.101	-3.10
**Succinate**	**0.665**	**0.008**	**0.98**	4-Aminobutyrate	0.662	0.082	-0.35	**Carnitine**	**0.717**	**0.048**	**-1.43**
**Threonine**	**0.664**	**0.003**	**1.03**	Homocysteine	0.658	0.093	-0.55	Glutamine	0.714	0.202	-1.89
**Glycine**	**0.658**	**0.004**	**0.87**	**Serine**	**0.654**	**0.040**	**-0.52**	Fumarate	0.712	0.056	-3.20
**Creatine**	**0.658**	**0.036**	**1.88**	**Ascorbate**	**0.645**	**0.044**	**-0.32**	Hypoxanthine	0.700	0.166	-3.04
**Glutamate**	**0.653**	**0.040**	**1.01**	Phenylalanine	0.645	0.853	0.49	Lactate	0.698	0.157	-1.88
**Carnitine**	**0.649**	**0.002**	**1.83**	Histidine	0.641	0.283	0.63	O-Phosphocholine	0.671	0.294	-2.75

**Histamine**	**0.649**	**0.007**	**2.51**	O-Phosphoethanolamine	0.637	0.430	-0.08	Histamine	0.657	0.190	-3.34
**Aspartate**	**0.648**	**0.033**	**-0.95**	Tyrosine	0.632	0.972	0.39	Phenylalanine	0.657	0.782	-1.98
**Methionine**	**0.645**	**0.035**	**1.26**	**Glutathione**	**0.628**	**0.035**	**-0.35**	Aspartate	0.652	0.151	-1.32
Histidine	0.643	0.143	2.43	Alanine	0.628	0.188	-0.13	Homocysteine	0.648	0.150	-2.97
**Guanosine**	**0.635**	**0.021**	**0.66**	Citrate	0.620	0.276	0.44	Cystathionine	0.645	0.316	-0.21

**Comparison IV: *IDH1*-mut Astro (n = 26) vs. *IDH1*-mut Oligo (n = 29)**				**Comparison V: *IDH1*-mut GB (n = 7) vs. *IDH1*-mut Astro (n = 26)**				**Comparison VI: *IDH1*-mut GB (n = 7) vs. *IDH1*-mut Oligo (n = 29)**				**Comparison VII: *IDH1*-wt Astro (n = 9) vs. *IDH1*-wt GB (n = 30)**			
**Biomarker**	**AUC**	**t-test**	**log FC2**	**Biomarker**	**AUC**	**t-test**	**log FC2**	**Biomarker**	**AUC**	**t-test**	**log FC2**	**Biomarker**	**AUC**	**t-test**	**log FC2**
**Carnitine**	**0.786**	**0.001**	**-3.95**	**Citrate**	**0.951**	**0.004**	**5.90**	**Sarcosine**	**0.966**	**7.23E-04**	**-8.01**	**scyllo-Inositol**	**0.785**	**4.86E-05**	**2.89**
**O-Phospho-ethanolamine**	**0.744**	**0.005**	**-1.99**	**Histamine**	**0.901**	**0.008**	**5.74**	**Glutamate**	**0.926**	**0.004**	**-6.26**	**4-Amino-butyrate**	**0.778**	**0.001**	**2.88**
**Ethanolamine**	**0.728**	**0.032**	**-2.37**	**Glutathione**	**0.896**	**2.47E-04**	**2.72**	**Taurine**	**0.906**	**0.020**	**-6.94**	**Alanine**	**0.778**	**0.028**	**0.18**
**scyllo-Inositol**	**0.707**	**0.021**	**-2.12**	**Formate**	**0.879**	**0.015**	**5.06**	**AMP**	**0.892**	**9.62E-05**	**0.83**	Betaine	0.774	0.227	2.37
**Histidine**	**0.696**	**0.007**	**-0.77**	**Uridine**	**0.868**	**0.002**	**5.50**	**Uridine**	**0.892**	**4.16E-04**	**-7.83**	Phenylalanine	0.759	0.113	0.36
**Sarcosine**	**0.695**	**0.014**	**-3.00**	**AMP**	**0.863**	**3.46E-04**	**-1.31**	**Phenylalanine**	**0.887**	**1.57E-05**	**-3.84**	Tyrosine	0.744	0.149	0.47
Guanosine	0.684	0.147	0.84	Acetate	0.857	0.062	4.80	**Lactate**	**0.887**	**1.60E-04**	**-4.31**	Valine	0.737	0.145	0.51
**GSSG**	**0.682**	**0.010**	**-0.78**	**Histidine**	**0.846**	**0.006**	**5.51**	**Glutathione**	**0.887**	**8.05E-04**	**-3.77**	**myo-Inositol**	**0.722**	**7.54E-04**	**2.61**
**Nicotinurate**	**0.676**	**0.029**	**-0.23**	**Glutamate**	**0.830**	**0.023**	**4.66**	Formate	0.882	0.133	-6.75	**Creatine**	**0.711**	**0.006**	**2.03**
**Citrate**	**0.674**	**0.035**	**-0.77**	**Taurine**	**0.819**	**0.003**	**5.18**	**Citrate**	**0.872**	**0.019**	**-6.66**	Hypotaurine	0.704	0.194	-0.28
Lactate	0.662	0.113	-1.08	**Butyrate**	**0.813**	**0.009**	**2.60**	**Succinate**	**0.852**	**0.001**	**-4.33**	Histidine	0.704	0.273	2.82
**O-Acetylcholine**	**0.658**	**0.030**	**-1.93**	**Lactate**	**0.791**	**0.019**	**3.23**	**Butyrate**	**0.837**	**0.006**	**-4.11**	**Hypoxanthine**	**0.696**	**0.007**	**2.38**
**Mannitol**	**0.639**	**0.033**	**-0.91**	**Serine**	**0.780**	**0.046**	**3.00**	Histidine	0.818	0.063	-7.10	**Sarcosine**	**0.696**	**0.040**	**3.30**
Acetate	0.634	0.954	-1.02	**O-Acetylcholine**	**0.775**	**0.013**	**2.89**	**Tyrosine**	**0.808**	**3.91E-04**	**-4.22**	Ethanolamine	0.685	0.797	2.76
Glutathione	0.633	0.727	-1.05	**Phenylalanine**	**0.769**	**0.024**	**2.86**	**Mannitol**	**0.808**	**0.006**	**-4.10**	**Creatine**	**0.682**	**0.024**	**2.55**

												**phosphate**			
**Taurine**	**0.629**	**0.038**	**-1.76**	**Succinate**	**0.764**	**0.025**	**3.10**	Methionine	0.803	0.120	-4.40	Acetate	0.682	0.102	2.06
O-Phosphocholine	0.627	0.272	-1.80	GTP	0.764	0.064	5.34	**Histamine**	**0.793**	**0.020**	**-6.55**	Cystathionine	0.678	0.060	-0.11
myo-Inositol	0.621	0.160	-0.93	4-Aminobutyrate	0.758	0.070	4.78	scyllo-Inositol	0.783	0.122	-6.25	**Ascorbate**	**0.674**	**0.028**	**2.63**
**Phenylalanine**	**0.615**	**0.047**	**-0.98**	N-Acetylaspartate	0.753	0.092	5.23	**Isoleucine**	**0.778**	**7.36E-04**	**-3.88**	Leucine	0.674	0.237	0.68
GTP	0.614	0.112	-0.25	Inosine	0.742	0.070	4.86	**Glucose**	**0.768**	**0.033**	**-4.17**	Isoleucine	0.667	0.317	0.45
2-Hydroxybutyrate	0.614	0.133	-1.01	**Fumarate**	**0.736**	**0.028**	**4.68**	**Serine**	**0.768**	**0.039**	**-4.54**	Glycine	0.663	0.171	0.81
Hypoxanthine	0.602	0.855	-0.45	Methionine	0.731	0.169	2.85	**Fumarate**	**0.749**	**0.046**	**-5.88**	O-Acetylcholine	0.656	0.268	0.87
3-Hydroxybutyrate	0.597	0.927	-1.60	Tyrosine	0.720	0.076	3.08	Carnitine	0.739	0.074	-5.93	Mannitol	0.652	0.116	0.97
Alanine	0.595	0.311	-1.57	Hypoxanthine	0.703	0.188	4.54	4-Aminobutyrate	0.739	0.078	-6.26	GTP	0.648	0.153	1.97
Glutamate	0.594	0.311	-1.60	Isoleucine	0.703	0.311	2.96	**Valine**	**0.724**	**0.006**	**-4.55**	Uridine	0.648	0.732	2.19

Supplementary table ST1: Top 25 AUC details for univariate ROC biomarker analysis of comparisons I-III in the "*IDH1* mutation status cohort", and comparisons IV - VII for different histologies and identical *IDH1* status in the "*IDH1* mutation status cohort". Abbreviations: AMP, adenosine monophosphate; Astro, astrocytoma; AUC, area under curve; Dex, dexamethasone; FC, fold change; GB, glioblastoma; GTP, guanosine triphosphate; GSSG, glutathione disulfide; Oligo, oligodendroglioma; ROC, receiver operator characteristics; Explanations: Bold are biomarkers with $p < 0.05$; Highlighted in grey are biomarkers shown and discussed in the manuscript and correlated with gene expression data.

Supplementary Table ST2: Basic clinical characteristics of study population

ID	*IDH1* status	Treatment prior to tissue acquisition	Histo	WHO	Age (years)	Resection	PFS (months)	OS (months)
1201	*IDH1*-wt	**Untreated**	GB	IV	50	1	12	18
1202	*IDH1*-wt	**Untreated**	GB	IV	78	1	13	19
1203	*IDH1*-wt	**Untreated**	GB	IV	65	1	3	13
1204	*IDH1*-wt	**Untreated**	GB	IV	49	1	11	no information
1205	*IDH1*-wt	**Untreated**	GB	IV	45	1	6	10
1206	*IDH1*-wt	**Untreated**	GB	IV	70	1	7	15
1207	*IDH1*-wt	**Untreated**	GB	IV	45	1	4	28
1208	*IDH1*-wt	**Untreated**	GB	IV	55	1	11	35
1209	*IDH1*-wt	**Untreated**	GB	IV	70	1	4	12
1210	*IDH1*-wt	**Untreated**	Astro	III	49	1	6	18
1211	*IDH1*-wt	**Untreated**	GB	IV	47	1	4	37
1214	*IDH1*-wt	**Untreated**	GB	IV	48	1	> 53	n/a
1215	*IDH1*-wt	**Untreated**	GB	IV	58	1	14	20
1218	*IDH1*-wt	**Untreated**	GB	IV	71	1	4	12
1219	*IDH1*-wt	**Untreated**	GB	IV	72	1	12	no information
1220	*IDH1*-wt	**Untreated**	GB	IV	80	1	6	13
1221	*IDH1*-wt	**Untreated**	Astro	II	37	1	no information	no information
1222	*IDH1*-wt	**Untreated**	GB	IV	77	1	no information	no information
1224	*IDH1*-wt	**Untreated**	GB	IV	70	1	6	13
1225	*IDH1*-wt	**Untreated**	GB	IV	71	1	no information	no information
1226	*IDH1*-wt	**Untreated**	Astro	II	40	1	> 36	n/a
1227	*IDH1*-wt	**Untreated**	GB	IV	50	1	16	31
1228	*IDH1*-wt	**Untreated**	GB	IV	77	1	no information	no information
1401	*IDH1*-mut	**Untreated**	GB	IV	33	1	56	n/a
1402	*IDH1*-mut	**Untreated**	GB	IV	34	1	> 56	n/a
1403	*IDH1*-mut	**Untreated**	GB	IV	40	1	> 55	n/a
1404	*IDH1*-mut	**Untreated**	Astro	III	40	1	> 52	n/a
1405	*IDH1*-mut	**Untreated**	Astro	III	45	1	36	> 57
1406	*IDH1*-mut	**Untreated**	Astro	III	32	1	> 60	n/a
1407	*IDH1*-mut	**Untreated**	GB	IV	37	1	31	> 63
1408	*IDH1*-mut	**Untreated**	Oligo	II	56	1	11	> 27
1409	*IDH1*-mut	**Untreated**	GB	IV	48	1	no information	no information
1410	*IDH1*-mut	**Untreated**	GB	IV	34	1	> 48	no information
1411	*IDH1*-mut	**Untreated**	Astro	III	34	1	15	> 50
1412	*IDH1*-mut	**Untreated**	Oligo	II	71	1	> 52	n/a

1414	*IDH1*-mut	**Untreated**	Oligo	II	32	1	> 48	n/a
1416	*IDH1*-mut	**Untreated**	Oligo	II	57	1	> 39	n/a
1417	*IDH1*-mut	**Untreated**	Astro	II	63	1	> 57	no information
1418	*IDH1*-mut	**Untreated**	Astro	II	45	1	no information	no information
1419	*IDH1*-mut	**Untreated**	Astro	II	36	1	> 40	n/a
1420	*IDH1*-mut	**Untreated**	Astro	II	62	1	41	> 52
1424	*IDH1*-mut	**Untreated**	Astro	II	39	1	76	> 88
1427	*IDH1*-mut	**Untreated**	GB	IV	34	1	no information	no information
1429	*IDH1*-mut	**Untreated**	Oligo	II	47	1	> 36	n/a
1430	*IDH1*-mut	**Untreated**	Astro	III	28	1	15	no information
2726	*IDH1*-mut	**Untreated**	Oligo	III	43	1	25	156
3088	*IDH1*-mut	**Untreated**	Oligo	III	45	2	25	156
1506	*IDH1*-mut	**Untreated**	Oligo	II	66	1	73	103
2686	*IDH1*-mut	**Untreated**	Oligo	III	71	2	73	103
5096	*IDH1*-mut	**Untreated**	Oligo	II	45	1	32	48
5939	*IDH1*-mut	**Untreated**	Oligo	III	48	2	32	48
2287	*IDH1*-mut	**Untreated**	Oligo	II	29	1	23	175
2754	*IDH1*-mut	**Untreated**	Oligo	III	31	2	23	175
3509	*IDH1*-mut	**Untreated**	Astro	II	37	1	31	83
4133	*IDH1*-mut	**Untreated**	Astro	III	39	2	31	83
5120	*IDH1*-mut	**Untreated**	Astro	II	22	1	13	45
5475	*IDH1*-mut	**Untreated**	Astro	III	23	2	13	45
T01	*IDH1*-mut	**Untreated**	Astro	II	39	1	76	> 86
T02	*IDH1*-mut	**Untreated**	Astro	II	39	2	76	> 84
T05	IDH1-wt	**Untreated**	Astro	III	45	1	35	> 51
T06	IDH1-wt	**Untreated**	Astro	III	45	2	no information	no information
1534	*IDH1*-wt	**RT**	Astro	III	51	1	14	18
1774	*IDH1*-wt	**RT**	Astro	III	52	2	14	18
2252	*IDH1*-wt	**RT -> PCV**	Astro	III	47	1	16	18
2581	*IDH1*-wt	**RT -> PCV**	Astro	III	48	2	16	18
4589	*IDH1*-mut	**RT**	Astro	III	24	1	42	68
5647	*IDH1*-mut	**RT**	Astro	III	37	2	42	68
3071	*IDH1*-mut	**RT**	Astro	III	35	1	66	73
4218	*IDH1*-mut	**RT**	Astro	III	39	2	66	73
3186	*IDH1*-mut	**RT**	Astro	III	29	1	60	116
4306	*IDH1*-mut	**RT**	Astro	III	34	2	60	116
3648	*IDH1*-mut	**RT -> TMZ**	Astro	III	36	1	54	110

4942	*IDH1*-mut	**RT ->TMZ**	Astro	III	40	2	54	110
T03	*IDH1*-mut	**RT -> TMZ**	Astro	III	39	1	10	> 74
T04	*IDH1*-mut	**RT -> TMZ**	Astro	III	39	2	10	> 72
T07	*IDH1*-wt	**RT/TMZ->TMZ**	GB	IV	31	1	9	30
T08	*IDH1*-wt	**RT/TMZ->TMZ**	GB	IV	31	2	9	30
T09	*IDH1*-wt	**RT/TMZ->TMZ**	GB	IV	39	1	5	39
T10	*IDH1*-wt	**RT/TMZ->TMZ**	GB	IV	39	2	5	39
T11	*IDH1*-wt	**RT/TMZ->TMZ**	GB	IV	54	1	6	12
T12	*IDH1*-wt	**RT/TMZ->TMZ**	GB	IV	54	2	6	12
T13	*IDH1*-wt	**RT/TMZ->TMZ**	GB	IV	53	1	9	18
T14	*IDH1*-wt	**RT/TMZ->TMZ**	GB	IV	53	2	9	18
T15	*IDH1*-wt	**RT/TMZ->TMZ**	GB	IV	50	1	9	20
T16	*IDH1*-wt	**RT/TMZ->TMZ**	GB	IV	50	2	9	20
1742	*IDH1*-mut	**TMZ**	Oligo	III	36	1	68	152
2875	*IDH1*-mut	**TMZ**	Oligo	III	41	2	68	152
3663	*IDH1*-mut	**TMZ**	Oligo	III	33	1	54	97
4943	*IDH1*-mut	**TMZ**	Oligo	III	38	2	54	97
3637	*IDH1*-mut	**TMZ**	Oligo	III	47	1	78	110
5446	*IDH1*-mut	**TMZ**	Oligo	III	53	2	78	110
3346	*IDH1*-mut	**TMZ**	Oligo	III	31	1	114	133
5837	*IDH1*-mut	**TMZ**	Oligo	III	40	2	114	133
2396	*IDH1*-mut	**RT->TMZ**	Oligo	III	33	1	30	52
2913	*IDH1*-mut	**RT->TMZ**	Oligo	III	35	2	30	52
1991	*IDH1*-mut	**RT**	Oligo	III	58	1	60	95
2930	*IDH1*-mut	**RT**	Oligo	III	63	2	60	95
5086	*IDH1*-mut	**RT->PC**	Oligo	III	57	1	16	23
5527	*IDH1*-mut	**RT->PC**	Oligo	III	58	2	16	23
2065	*IDH1*-mut	**RT**	Oligo	III	36	1	56	221
2996	*IDH1*-mut	**RT**	Oligo	III	40	2	56	221

Supplementary Table ST2: Basic clinical characteristics of study population. Patients have been characterized by their assigned identity number (ID), *IDH1* mutation status, treatment prior to tissue acquisition, histology and WHO grade (according to WHO classification 2016), age, resection status, PFS and OS. Abbreviations: Astro, Astrocytoma; GB, Glioblastoma; Histo, Histology; ID, Identifier; Oligo, oligodendroglioma; OS, overall survival; PC, procarbacine/lomustine; PCV, procarbazine/lomustine/vincristine; PFS, progression free survival; RT, radiotherapy; TMZ, temozolomide; WHO, World Health Organization; Further details on cohorts are outlined in Figure 1 and in the text.
